# Proline stabilizes amphiphilic gold nanoparticles *via* hydrophobic interactions

**DOI:** 10.1039/d5nh00260e

**Published:** 2025-09-16

**Authors:** Ting Mao, Quy Ong, Joachim Kohlbrecher, Ekaterina Poliukhina, Paulo Jacob Silva, Francesco Stellacci

**Affiliations:** a Institute of Materials, Ecole Polytechnique Fédérale de Lausanne (EPFL) Lausanne Switzerland francesco.stellacci@epfl.ch; b PSI Center for Neutron and Muon Sciences, Paul Scherrer Institute Villigen PSI CH-5232 Switzerland; c Bioengineering Institute, Ecole Polytechnique Fédérale de Lausanne (EPFL) Lausanne Switzerland

## Abstract

Colloidal dispersions are key in many fields of science and technology. Recently, we have shown that small molecules can stabilize dispersions of nanoscale objects, such as proteins and nanoparticles by screening their net attractive interactions. This new effect is essentially the opposite of the well-known salt screening of electrostatic interaction. Here we show that small molecule stabilization of nanoparticles is a phenomenon strongly linked to the hydrophobic content of the particles as well as to the strength of their hydrophobic attraction. We compare the effect of proline on gold nanoparticles coated with 11-mercaptoundecane sulfonate (MUS) at varying percentages of the hydrophobic ligand octanethiol (OT). We show that the larger the percentage of OT, the larger the proline stabilization effect is. We also compare the effect of proline on water dispersions of nanoparticles with that on heavy water dispersions. In the latter, the hydrophobic effect plays a bigger role. We find that in D_2_O, proline stabilization is larger. We also compare the effect of proline on the same MUS:OT gold nanoparticles before and after an annealing process that is known to render the particle more hydrophilic. Proline is more effective on the particles before annealing. Finally, we study the effect of proline on non-aggregating allMUS nanoparticles. We find that proline stabilization of these particles is mainly due to a reduction in the long-range attraction coefficient. Overall, we show that proline stabilizes nanoparticle dispersions more effectively as the hydrophobic attraction between nanoparticles increases.

## Abstract 2

Proline stabilizes gold nanoparticle dispersions. Using gold nanoparticles as a model system, we identify the scale, magnitude, and nature of the screening effect. Screening becomes stronger as the hydrophobic attraction between particles increases.

## Graphical abstract

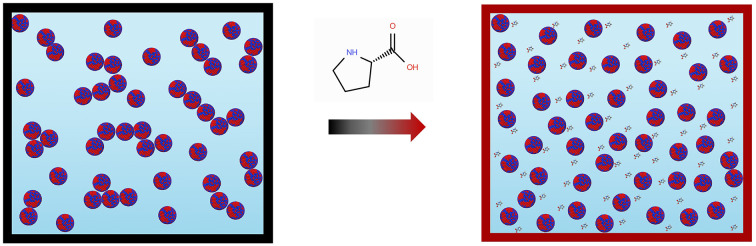

**New concepts**

Recently, we have introduced the concept of colloidal stabilization of nanoscale objects, such as nanoparticles or proteins, by the addition of small molecules, including amino acids. Here we focus on gold nanoparticle coated by a mixture of hydrophobic and hydrophilic ligands and show that proline, an amino acid, significantly stabilizes their water dispersions. We find that the stronger the hydrophobic interactions between the particles the stronger the stabilization is.

## Introduction

The stability of a dispersion of colloids^1,2^ is typically explained by the Derjaguin–Landau–Verwey–Overbeek (DLVO) theory with a balance between electrostatic repulsive forces and van der Waals/electrostatic attractive forces.^3–6^ While classical DLVO theory is able to correctly describe most dispersions of charged colloids, various extended DLVO (xDLVO) theories have been developed to address some more complex cases.^7–10^ In the case of dispersions of small colloids (*e.g.* nanoparticles smaller than 10 nm in diameter or proteins), concerns have been raised about key assumptions of the theory such as the additivity of the interactions,^11–14^ or the solvent molecules and/or ions being negligible in size relative to colloids.^12,14–17^ Despite these limitations, DLVO theory remains a foundational framework for understanding colloidal dispersions.

The stability of colloidal dispersions plays a critical role in diverse applications such as pharmaceutical formulations,^18,19^ controlled self-assembly for protein crystallization,^20,21^ and targeted drug delivery.^22,23^ Within DLVO theory's framework, in order to increase or decrease the stability of a colloidal dispersion, one must modulate the colloidal interactions. It is well known that repulsive interactions can be modulated for example by adjusting the ionic strength of the solvent^6^ or altering the solution pH.^24^ Much less is known about how to reduce colloids’ attraction.^25,26^ Recently, we have presented experimental results showing that small molecules (SMs), and especially amino acids (AAs), can modulate the net colloidal attraction in dispersions of nanoscale objects such as gold nanoparticles as well as proteins.^27^ Briefly, by studying the second osmotic virial coefficient and the potential of mean force (PMF) of dispersions either of proteins, or of gold nanoparticles, we have shown that the addition of small molecules reduces the effective attraction. Specifically, the second virial coefficient (B_22_) of the colloids increases as the concentration of small molecules increases in a Langmuir fashion. By analysing the experimental results, we developed the hypothesis that SMs interact weakly with nanoscale objects (NOs) leading to an effective screening of nearest neighbour interactions. We consequently developed a theoretical framework that relates the changes in B_22_ with the concentration and the disassociation constant (*K*_D_) of the SMs to NOs. We validated the theory by comparing the experimentally measured *K*_D_ to the ones derived by fitting the experimental data about the increase in B_22_ with the formula we proposed. We found that the two values found for *K*_D_ were never more than a factor of two apart. We also validated our theory by experimentally proving some of its predictions, for example that short peptides would stabilize colloidal dispersions of protein nanoparticles equally well or better than AAs. Because of these findings, we have discovered that AA addition to dispersions leads to significant changes in protein–protein self- and cross-interactions already at the millimolar scale, or, at larger concentrations, it leads to changes in cell's stress granule formations, viral replication, or bioavailability of insulin in mice experiments.^27^

While our theoretical framework explains the observed effects by assuming that small molecules screen a fraction of the attractive interactions among the colloids, the specific nature of such interactions affected has remained unclear.

In this study, we aim to investigate which colloidal interactions are influenced by small molecule additives. To achieve this goal, we focus on studying the effect of proline on the colloidal interactions of ligand-coated gold nanoparticles. We use proline as it is the primary small molecule studied in our previous work, and it shows similar stabilization effects on both nanoparticles and proteins.^27^ We focus here on nanoparticles as model systems because compared to proteins, their surface properties can be easily tuned, and the determination of their PMFs is more straightforward. The hypothesis we test in this paper is that proline modulates nanoparticles’ attractive interactions and this modulation scales with nanoparticle hydrophobicity. To test such hypothesis, we modulated the particles’ hydrophobicity in 3 distinct ways. We studied particles coated with a mixture of hydrophobic and hydrophilic ligands and varied their ratio. We used in-solution thermal annealing to change the ligand shell so as to vary the particles’ overall hydrophobicity. We replaced water with deuterated water known to increase hydrophobic attraction. In all three cases, as the particles became more hydrophobic, they showed a markedly increased tendency to aggregate confirming that hydrophobicity is the dominant attractive interaction among them. PMF results indicate that proline addition induced stronger stabilization effects when hydrophobicity of the nanoparticles increased. A careful study of the interaction potential of non-aggregating particles derived from small angle X-ray scattering with a 2-Yukawa potential showed that proline addition influences predominantly long-range attraction.

## Results

We choose gold nanoparticles (Au NPs) functionalized with different ratios of hydrophobic and hydrophilic ligands to control the hydrophobicity of the colloids. The hydrophobic ligand was OT, while the hydrophilic one was MUS. For our studies, it is advantageous to have nanoparticles monodisperse in size. To synthesize monodisperse MUS:OT Au NPs, we followed a two-step approach where oleylamine-functionalized monodisperse Au NPs were first synthesized and their ligands were replaced with MUS and OT following a literature procedure developed by Guldin and coworkers.^28^ When starting from a single large batch of oleylamine-coated Au NP, such an approach allows performing ligand replacement reactions at varying ligand ratios so to obtain particles with different ligand shell compositions but the same Au core. This advantage suits well the goal of this study where we compare the difference of interaction of nanoparticles by varying their surface chemistry while ruling out possible influences that could result from differences in the size of the core. In this study, we used Au NPs with an average size of 3.8 nm and a standard deviation of 0.4 nm, as derived from transmission electron microscopy (TEM) (see Fig. S1). This characterization agrees well with the size of 3.5 nm determined by the fitting of small angle X-ray scattering (SAXS) data (see Fig. S10). We used three Au NPs, differing in the ligand shell composition. Specifically, they differed in the percentage of OT ligands; they have 0% (allMUS), 21% (21OT) and 44% (44OT) OT in their ligand shell. Calculation of the ligand shell composition was performed using nuclear magnetic resonance (NMR) spectroscopy after the decomposition of the Au core (Fig. S3). Before the decomposition, all particles were deemed to be free of unbound ligands as their NMR spectra showed no sharp peaks (Fig. S2).^29^

**Fig. 1:**
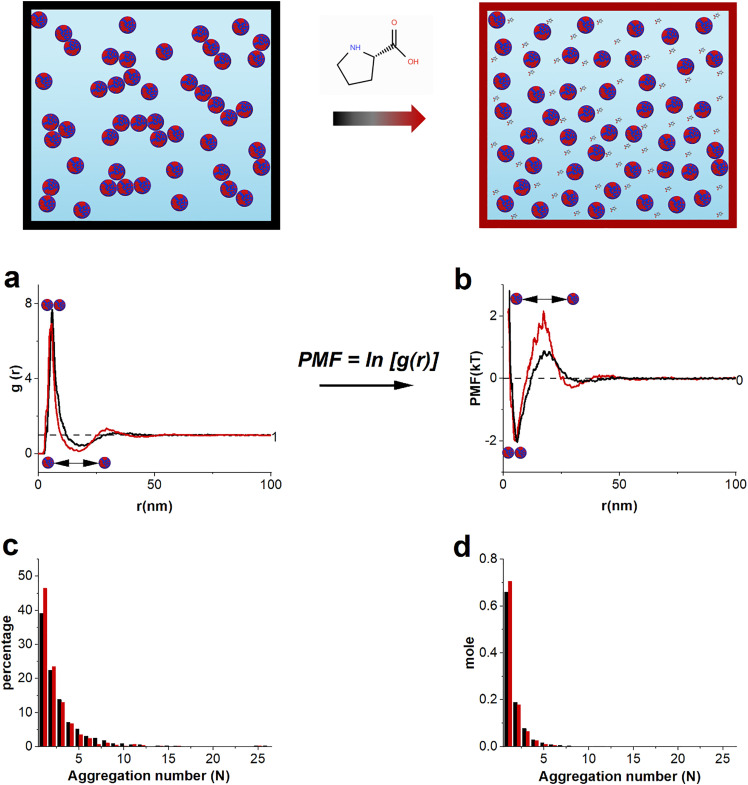
The top cartoons illustrate the effect of small molecules on aggregating system. The black box indicates the water dispersion of the 44OT AuNPs in their aggregating form in water, while with the addition of small molecules (proline), the aggregations are being dispersed (red box). (a) and (b) depict the RDF and PMF of the 44OT AUNPs, respectively, of the small molecule effect, comparing water with or without proline at similar number concentrations (in units of particles per m^3^): the black curve indicates the 44OT AuNPs in water, 6.39 × 10^22^. The red curve indicates the 44OT AuNPs in water with the addition of 2 M proline, 7.29 × 10^22^. (c) and (d) are histograms representing the percentage (c) and mole fraction (d) of the aggregate present in water (black) and water with 2M proline (red).

In an n-body system, a particle experiences a mean force that arises from the instantaneous interactions with the rest of the particles in the system and the PMF is defined as a free energy function whose negative gradient with respect to the relative coordinates of the particles yields this mean force.^30^ The PMF –*W*(*r*)– is related to the n-particle probability distribution function or center of mass radial distribution function (RDF, *g*(*r*)) by*W*(*r*) = −*kT* ln[*g*(*r*)]where *k* is the Boltzmann constant and *T* is the absolute temperature. To obtain the PMF of these Au NPs in their dispersions in aqueous solutions, we employed the RDF obtained using the particles’ centroids positions from the 3D particle distribution derived from cryogenic transmission electron microscopy (Cryo-TEM) tomography. We used a workflow that we recently developed and made use of the fact that in ref. 31, we showed that the particle distribution in the vitrified state is the same as in the distribution in aqueous dispersion. An example of conversion of the RDF of the 44OT particles to their PMF is shown in Fig. 1. Image analysis provided another important information on this sample, *i.e.* the aggregation states of the particles (Fig. 1(c) and (d)). The key difference between the percentage histogram and the mole one lies in how aggregation states are represented. The percentage histogram shows the percentage of individual particles involved in various aggregation states (see the Materials and methods section). In contrast, the mole histogram represents the fraction of each aggregation state, where an aggregate consisting of N particles is counted as a single entity. While both of these representations are informative about aggregation states, in the following text, we only refer to the percentage histogram for simplicity.

In Fig. 1(a) and (b), we also show the RDF and PMF of the 44OT AuNPs when 2 M proline is added. These are the sample data and plots shown in ref. 27. Both the RDF and PMF of 44%OT in water with or without proline have similar features. The peak in the RDF at ∼7 nm indicates that this system contains a good number of aggregates given that the particle diameter is approximately this value. The aggregation of these particles is essentially the particles touching each other. At a further distance (∼20 nm), there is a minimum of the RDF, indicating that particle density is depleted. At ∼35 nm, there is a subtle broad peak, indicating a slight preference of particles for this relative distance. The corresponding PMF is shown in Fig. 1(b). At 7 nm, we now find an energy minimum in PMF, while at 20 nm, it shows an energy barrier that particles must overcome in order to form aggregates. At 35 nm, there is a shallow minimum. The energy difference between the 7 nm energy well and its barrier at 20 nm is only 4 *kT*, indicating a rather dynamic system with particles that associate and dissociate often. This fact is in agreement with our observation that dispersions of these particles are rather stable over time, despite the relatively high fraction of aggregating particles. In fact, we have measured the SAXS spectra for solutions of these particles for a period of one month. In Fig. S4 one can easily note that the spectra are basically unchanged over time. The features discussed in the PMF of the 44OT AuNPs are consistent with recent literature studies.^32^ The addition of proline (red curves, in Fig. 1) increases the energy barrier for aggregation and slightly lowers the energy well. This in turn changes the aggregation state of the particles by increasing the monomer population by 8% and the dimer population by 1%, while noticeably decreasing the populations of all the higher aggregation numbers.

To investigate the role of hydrophobic content on the colloidal interactions, we used three approaches. First, we varied the ligand shell composition. Second, we kept the composition constant but we changed the morphology of the ligand shell so as to change the wetting of the particles using a method described in ref. 33 and 34. Third, we replaced light water with heavy water to increase the hydrophobic attraction among the particles.^35^

**Fig. 2:**
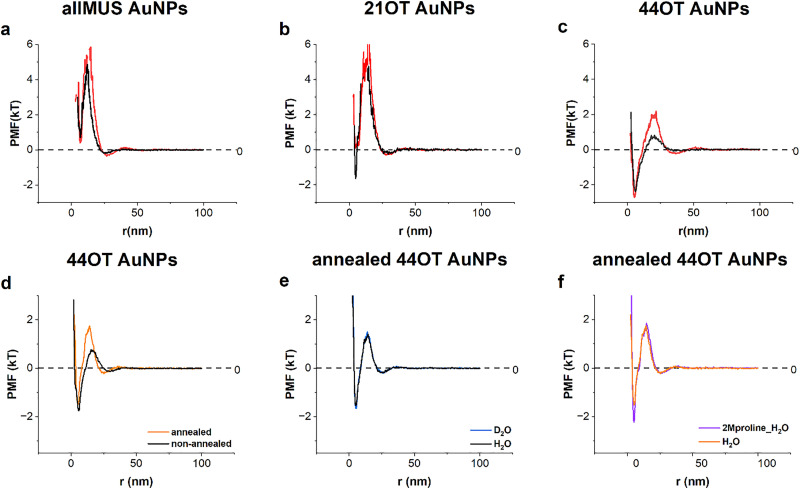
Proline stabilization effect in AuNPs systems with varying hydrophobicity. Number concentrations, in units of particles per m^3^. (a) allMUS AuNPs in water, at a number concentration of 5.28 × 10^22^ (black), compared with the addition of 2 M proline at a number concentration of 5.21 × 10^22^ (red); (b) 21OT AuNPs in water, at a number concentration of 4.24 × 10^22^ (black), compared with the addition of 2 M proline at a number concentration of 4.36 × 10^22^ (red); (c) 44OT AuNPs in proline, at a number concentration of 4.89 × 10^22^ (red), compared with the addition of water at a number concentration of 5.14 × 10^22^ (black). Effect of annealing in the PMF of 44OT AuNPs. Number concentrations, in units of particles per m^3^. (d) PMF of 44OT in water (11.35 × 10^22^ (black)), annealed 44OT for 24 hrs (11.6 × 10^22^ (orange)). (e) Annealed 44OT in light (11.6 × 10^22^ (black)) and heavy water (12.2 × 10^22^ (blue)); (f) annealed 44OT in light water (10.6 × 10^22^ (orange)) and with addition of 2 M proline (9.8 × 10^22^ (purple)).

The first approach consisted in changing the ligand shell composition of the particles. As expected, as the OT percentage increased, the particles became more hydrophobic as confirmed by an increase in the aggregation state. The monomer percentage went from 96% in allMUS to 89% in 21OT, to 35% in 44 OT (see Fig. S9). In Fig. 2(a)–(c), we show the PMF for allMUS, 21OT, and 44OT particles. The curves in black represent the particles in water, and the ones in red represent the particles at the approximately same number density but with 2 M proline added. The number densities are also approximately the same for the different particle systems. The role of number density on the PMF and the PMF change as a function of proline has been discussed in ref. 27. Briefly as the number density decreases, the effect of proline decreases as the interparticle interactions become weaker. The largest changes are observed for the 44OT particles, while for the 21OT particles, the only noticeable change is that the potential well becomes much less deep and in fact raises above zero. For the allMUS particles, insignificant change can be observed with the exception that the width of the potential barrier becomes larger; in other words, particles start repelling each other at larger distances. These data are corroborated by the aggregation plots for these particles (see Fig. S7). For the 44OT, we find that proline increases drastically the monomer population by around 10% and the dimer population by around 3%, while noticeably decreasing the populations of all higher-number aggregates. Importantly, for the 21OT as the potential well moved slightly above zero, when proline is present, we see the monomer fraction going from 90% to 93%. For allMUS, the whole sample is in the same monomeric form (96%) with and without proline. In all cases, our data indicate clearly that proline's stabilization effect is much stronger than the hydrophobic nature of the particles.

Mixed ligand NP systems are known to form patch-like domains on their surface.^36^ The thermodynamic origin of such domain formation is the competition between the entropic gain of intermixing of dissimilar ligands and the enthalpy driven for phase separation.^36,37^ External perturbation like thermal annealing can change patterns on the surface, bringing the morphology closer to its equilibrium state. In the study by Zhi *et al.*^33^, we showed that Au NPs coated with a 1 : 1 binary mixture of 3-mercaptopropionic acid (d-MPA) and OT upon thermal annealing change their ligand shell morphology and consequently change their wetting properties as indicated by a significant change in their work of adhesion. We decided to perform thermal annealing on 44OT in an attempt to achieve the same effect. We heated these particles at 50 °C for 24 h. NMR spectra showed that the ligand shell composition did not change upon annealing (see Fig. S5), while analytical ultracentrifugation studies proved that no change in size took place (see Fig. S6). The latter is also confirmed by the fact that the position of the potential well in the PMF does not change upon annealing (Fig. 2(d)). In stark contrast, the aggregation state of the particle changes significantly. In Fig. 2(d), we show the PMF of the 44OT particles before and after annealing. We notice that after annealing, there is a significant increase in the energy barrier by almost 1 *kT* and it also shows a shift of the barrier position to shorter distances. Overall, the shape of the PMF moves close to that of the 21OT particles, confirming that the particles became more hydrophilic. More interestingly, the small molecule stabilization effect of proline on the annealed 44OT is now minimal as shown in Fig. 2(f).

**Fig. 3:**
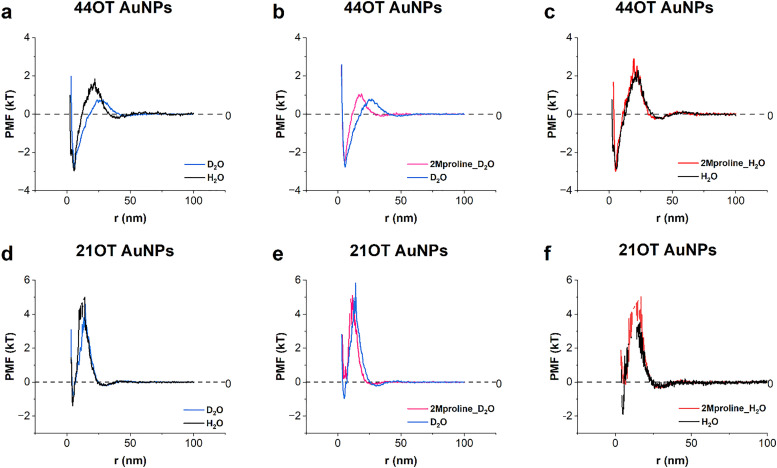
Isotope effect in two AuNP systems (44OT AuNPs (a)–(c) and 21OT (d)–(f)) with different hydrophobicities. Number concentrations, in units of particles per m^3^: (a) effect on PMF of 44OT AuNPs in light water (3.53 × 10^22^ (black)), and heavy water (3.45 × 10^22^ (blue)); (b) effect of addition of 2 M proline of 44OT AuNPs in heavy water, with proline (4.02 × 10^22^ (purple)), and heavy water (3.73 × 10^22^ (blue)); (c) effect of addition of 2 M proline of 44OT AuNPs in light water (water 3.61 × 10^22^ (black), with proline (3.51 × 10^22^ (red)); (d) effect on PMF of 21OT AuNPs in light water (4.17 × 10^22^ (black)), and heavy water (4.06 × 10^22^ (blue)); (e) effect of addition of 2 M proline of 21OT AuNPs in heavy water, with proline 3.39 × 10^22^ (purple), and heavy water 3.86 × 10^22^ (blue); (f) effect of addition of 2 M proline of 21OT AuNPs in light water (water 4.24 × 10^22^ (black), with proline 4.24 × 10^22^ (red)).

In order to change the strength of the hydrophobic effect while keeping the particles completely unchanged, we replaced light water with heavy water. The isotope effect of the hydrophobic effect was recently studied by N. Patil *et al.*^35^ and they reported a 20% increase in the effective attractive force between hydrophobic surfaces interacting through heavy water as compared to light water. We performed PMF measurement on 44OT, 21OT and the annealed 44OT particles in light and heavy water. In all cases, we compared these PMFs to the ones in the presence of 2 M proline. The results are shown in Fig. 3. It is immediately evident that the energy barrier of 44OT is brought down by almost 1 kT when water is changed into heavy water (Fig. 3(a)) confirming that the attraction between particles has increased. Also, the energy barrier shifts to larger distances consistent with stronger net attractive interactions that are felt at larger distances. As expected, these changes are much less pronounced for 21OT particles (Fig. 3(d)). In Fig. 3(b), we show the effect of addition of 2 M proline on the PMF of 44OT particles in heavy water. While the stabilization effect of proline is very pronounced, it is also true that it is very different from the effect on the same particles at the same number density in light water. When comparing Fig. 3(b) with Fig. 2(c), one notices that in light water, the main effect of proline is to increase the energy barrier, and in heavy water, it moves the energy barrier to shorter distances with a smaller increase in barrier height. When proline is added in heavy water to 21OT particles, we find a much smaller effect but also in this case we observed a shift of the energy barrier to shorter distances (Fig. 3(e)). The data presented in this figure are at lower number densities of particles when compared to the ones shown in Fig. 2. At these number densities in H_2_O, proline has little effect on the PMF. In Fig. 3(c), we show data already presented and discuss in ref. 27 about this aspect. It is remarkable how proline's stabilization effect becomes large in D_2_O at these concentrations. A similar consideration holds for 21OT particles, and proline's stabilization effect in D_2_O is small but noticeable, while it is absent in H_2_O.

### SAXS structure factor of allMUS AuNPs

The PMF is the key tool to investigate colloidal interactions in dispersions, but it is not the simplest representation of such interactions because of the complexity of multi-body interactions. The most direct potential to interpret is the pair interaction potential (PIP). The PMF is equivalent to the PIP but only when the system is under infinite dilution where many body interactions can be excluded.^38^ Furthermore, the PIP is the limit for dilute concentrations of the PMF only for non-aggregating systems. The Cryo-TEM tomography technique we developed is based on particles’ position statistics and thus does not allow for arbitrary dilutions. Consequently, to determine the effect of proline on the PIP, we then chose to investigate a non-aggregating nanoparticle system (allMUS) and studied it with SAXS. We performed SAXS on a series of dispersions of allMUS particles in water at varying concentrations with and without 2 M proline. We used a 2-Yukawa potential to fit the SAXS structure factor of the particles. The form factors for all the fittings here are fixed as a log norm size distribution function fitted from experimentally measured allMUS NPs at very low concentration (see Fig. S1). For charged particles, it is typical to use Yukawa-like potentials for their fitting.^39^ All the fittings here are performed using SASfit.^40^ The equations are shown below
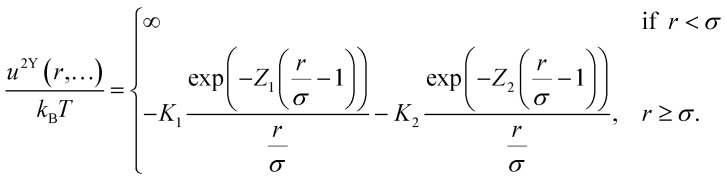
where *K* denotes the strength of the interaction, with *K* > 0 indicating attractive interactions; *Z* = 1/*λ* denotes the interaction range; *σ* is the diameter of the hard sphere.

**Fig. 4:**
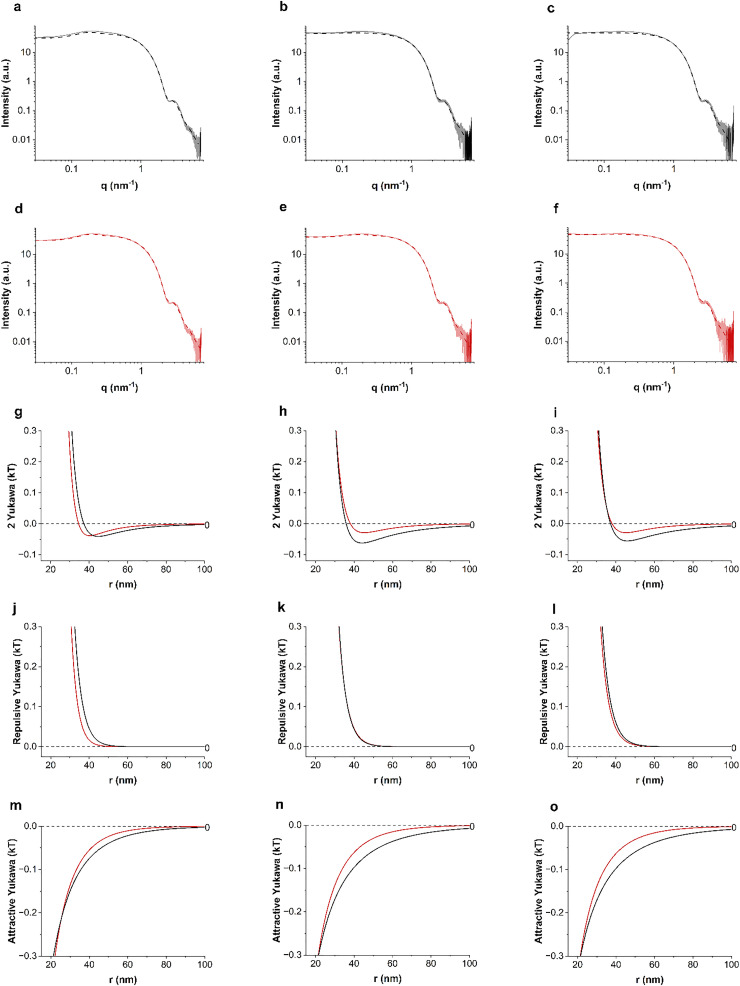
Fitted results from 2-Yukawa potential fitting on lower concentrations of allMUS AuNPs, 5 mg mL^−1^, 2.5 mg mL^−1^ and 1.25 mg mL^−1^. Interaction potentials of allMUS in water (a) and (b) and in 2 M proline (d)–(f). Proline's stabilization effect on the total 2-Yukawa potentials (g)–(i), repulsive Yukawa potentials (j)–(l), and attractive Yukawa potentials (m)–(o) of allMUS AuNPs shown in pair comparisons at the same weight concentrations.

In Fig. 4 we plotted the fitted total pair potential at the three lowest concentrations, comparing the pure water and water containing 2 M proline. The fitted 2-Yukawa potential changes when proline is added mostly at a distance larger than 30 nm (Fig. 4(g)–(i)). We decoupled the fitted 2-Yukawa potentials into attractive and repulsive potentials, as shown in Fig. 4. We note that most of the changes that proline induces are in the attractive potential (Fig. 4(m)–(o)) primarily through a reduction in attraction. Differences in the repulsive part of the potential can be observed only at higher nanoparticle concentrations, where, probably, the change in dielectric constant due to the addition of proline plays a role (see Fig. 4(j)–(l)). We measured the dielectric constant of the 2 M proline solution being 113, while that of pure water was 78.

## Discussion

Stabilization of colloidal dispersions by small molecules is a scientific topic of fundamental importance, particularly in the context of protein–protein interactions, where it has implications in the development of drug formulations.^19,41,42^ Numerous studies have been conducted to understand how these molecules stabilize proteins.^43–46^ It is well established that the hydrophobic effect is the driving force behind protein folding and self-assembly, and that certain small molecules stabilize the native folding of proteins.^47–49^ However, a clear correlation between small-molecule stabilization of proteins and their colloidal interactions has rarely been established.^50^ Furthermore, the specific interactions that are affected remains largely unreported.^51,52^ For example, Liu *et al.* explained the stabilizing effect of monohydric alcohol on lysozyme *via* an attractive square-well potential model without explaining what the origin of such attraction is.^51^ Similarly, Niebuhr *et al.* studied the effect of urea and TMAO on the interaction of lysozyme by fitting the SAXS structure factor, where they find that the effect of TMAO on lysozyme–lysozyme interactions can be explained by fixing the repulsive interaction while varying the attractive one, indicating the effect of TMAO on attractive interactions of lysozyme.^52^ The concentrations investigated in previous studies typically exceed 20 mg mL^−1^, which is relatively high compared to ideal interaction conditions. In this study, leveraging the high contrast of gold nanoparticles (AuNPs), we were able to measure colloidal interactions at low concentrations while still obtaining fit-worthy SAXS spectra. By focusing on the dilute regime, close to ideal pair-interaction conditions and without pre-assumed constraints on specific interactions, we successfully probed changes in attractive interactions caused by small molecules. The strength of this study lies in the ease of modulating the surface chemistry of AuNPs.

In our previous work,^27^ we hypothesized that small molecules screen interactions across various colloidal systems. We developed a framework to describe how changes in interactions among colloids depend on the concentration of small molecules. This approach proved valid by accurately capturing variations in the experimental second virial coefficient of colloids upon the addition of small molecules. It has shown that the same effect happens from many nanoscale colloidal objects, *i.e.* proteins, gold nanoparticles, and plasmid DNA.^27^ However, this theoretical framework does not specify which interactions are being modulated. In this study, we focused gold nanoparticles (AuNPs) as a model system, leveraging their tuneable surface chemistry. Additionally, AuNPs are thermodynamically stable, even in deuterated water, unlike proteins that often denature in heavy water. This stability allows us to correlate the long-range interactions observed in a repulsive system with hydrophobicity-induced aggregation in an aggregating system. We systematically investigated the effects of proline on AuNPs colloidal interactions using both direct measurements of the PMF and PIP derived from SAXS. We focused solely on proline as our previous work (ref. 27) showed that the effect of most amino acids is comparable, thus by studying proline we are confident that we have a good representation of the whole phenomenon.

The body of work that we have presented indicates that small molecules screen colloidal interaction by decreasing their net attractions. This can happen by increasing their repulsion or by decreasing their attraction. It is hard to envision how the former could be possible, hence we decided to test the latter in this work. The main attractive forces between colloids in water arise from van der Waals attraction and hydrophobic effect. In water, the latter leads to attraction among colloids. In this work, we tuned colloids’ hydrophobicity through three approaches: (1) modulating the surface ligand ratio on AuNPs between hydrophobic and hydrophilic ligands; (2) altering mixed-ligand patchiness *via* thermal annealing; and (3) using deuterated water as a solvent.

Fig. 2(a)–(c) presents three AuNP systems—0% OT (all MUS AuNPs), 21OT, and 44OT nanoparticles—that share the same gold core but differ in surface compositions, with varying ratios of hydrophobic to hydrophilic ligands. As expected, the particles become more hydrophobic with higher OT percentages, as evidenced by their change in aggregation state. AllMUS particles basically exist in the dispersion only in the monomeric state, while the 44OT is only at ∼30% in the monomeric state. As proline is added, changes in PMF are obvious for the 44OT particles and a lot more subtle for the other particles. For the 21OT, changes in the aggregation state are observed. These results show that increasing the hydrophobic content on the nanoparticle surface leads to stronger changes in the PMF and aggregation upon proline addition.

In our theory, the driving force for stabilization is the interactions of the particles with the small molecules.^27^ Based on this, we consider two possible interpretations for proline stabilization of AuNPS at varying ligand shell composition. One is that proline more efficiently screens the hydrophobic effect; the other is that proline binds selectively though not necessarily exclusively to the OT ligands. For this reason, we studied a system where the ligand shell composition (MUS : OT ratio) remains constant, but the ligand shell morphology (thus the hydrophobicity) changes due to annealing.^53^ In this system, the proline–ligand interactions should remain constant regardless of annealing, allowing us to isolate the effect of hydrophobicity. The NMR spectra confirmed that the ligand shell composition remained intact after annealing. Yet, the hydrophobicity of the particles changed significantly upon annealing as the pre-annealed particles have 47% monomers, while post-annealing, the monomeric fraction is 29%, as shown in Fig. S8. As discussed above, in the case of non-annealed 44OT particles, the addition of proline generates a very large change. For the annealed particles, this is no longer the case, and the nearly overlapping PMF in the presence of proline (Fig. 2(f)) highlights that the effect of proline is stronger with greater hydrophobicity and weaker with reduced hydrophobicity.

The hydrophobic effect is known to be stronger in heavy water (D_2_O) when compared to light water (H_2_O). Consistent with this, replacement of the solvent from H_2_O to D_2_O induced more aggregation in both 21%OT and 44%OT particles. This confirms that hydrophobic attraction is enhanced in D_2_O, as shown in the changes of PMF in Fig. 3(a) and (d). The stabilization effect of proline in heavy water is also more profound than the one in light water for both NPs (see Fig. 3). This is especially evident for the 21OT particles where proline shows a significant stabilizing effect only in D_2_O. Together, these findings reveal a direct relationship between particle hydrophobicity and proline's stabilization effect: the greater the hydrophobicity, the stronger the influence of proline.

To strengthen this conclusion, we further investigated the effect of proline on the PIP of allMUS particles. It is true that the proline's stabilization effect on allMUS particles as studied by PMF was smaller than what we could detect. But these particles are the only ones for which we could obtain a reliable PIP as they are the only ones that do not aggregate. The PIP was derived from a simple 2-Yukawa interaction potential fit (see Fig. 4) of the structure factor measured through SAXS. As expected, the proline's stabilization effect on these particles was small, but still detectable by this approach. We find that proline primarily reduces the long-range attraction, with minimal changes to the short-range repulsion. Although long-range attraction is a hallmark of hydrophobic interactions, it is not uniquely specific to them.^3,54^ Yet we attribute the proline-induced modulation of this long-range attraction to hydrophobic effects, as the same component increases upon the replacement of H_2_O with D_2_O, as shown in Fig. S11.

## Conclusions

In summary, we show that proline stabilizes gold nanoparticles more effectively as their attraction induced by the hydrophobic effect increases. Across all systems we studied, as the hydrophobicity of the particles increases, there is a decrease in the monomeric fraction of the particles, *i.e.* an increase in aggregations. The more the particles were aggregated the more proline was able to decrease such aggregation state and consequently stabilize the particles’ dispersion.

## Materials and methods

### Synthesis of nanoparticles

In a 500 mL three-neck round-bottom flask, 789.3 mg of HAuCl_4_·3H_2_O, 64 mL of oleylamine, and 80 mL of *n*-octane were mixed and stirred until complete dissolution of the solid. The flask was purged with argon for 10 minutes to establish an inert atmosphere. Subsequently, 16 mL of the *tert*-butylamine–borane complex (351.3 mg) dissolved in oleylamine was rapidly injected to initiate the reduction reaction. The mixture was continuously stirred for 1 hour, after which the reaction was quenched with 240 mL of ethanol. The resulting nanoparticles were precipitated *via* centrifugation. The crude nanoparticles were washed to remove the residual organic material: the precipitate was sonicated in fresh dichloromethane, followed by ethanol, and the processes of sonication, washing, and re-precipitation were repeated multiple times. After purification, 90 mg of oleylamine-capped nanoparticles were dissolved in 30 mL of dichloromethane. A solution of MUS, 34.86 mg and OT 31.23 μL was added to induce ligand exchange. The ligand exchange reaction was carried out under stirring in a sealed system for 21 hours. Prior to ligand-exchange, the nanoparticles were purified through repeated sonication and precipitation using fresh dichloromethane and acetone to remove residual organics. The resulting nanoparticles underwent further purification by dissolving them in ultrapure water and using Amicon® 30 kDa MWCO filters to eliminate excess MUS. The quality of purification and the final ligand composition of the nanoparticles were verified by NMR spectroscopy, as shown in (Fig. S2).

### Cryogenic transmission electron microscopy

3.5 μL of the dispersion was casted onto a previously glow discharged quantifoil grid (Quantifoil® R 1.2/1.3, 200 Mesh, Cu). The grid with solution was blotted with Whatman filter papers on both sides in a vitrobot (Vitrobot Mark IV) under 100% of humidity at 22 °C, followed by immediate vitrification in liquid ethane. Imaging was done at the Dubochet Center for Imagine (Lausanne) in a Titan Krios G4 operating at 300 kV. Tilt series were recorded from −60 to 60 with 2 increments at the magnification of 33000 X (camera pixel size of 0.37 nm) and a defocus of −7 μm with a total dose of 120 e Å^−2^. For data processing, pre-aligned.mrc files were compiled from Tomography version 5.16.0 (Thermo Fisher Scientific). The camera used was a Falcon 4i equipped with a Selectris X energy filter (slit width 20 eV).

The state of particles in monomeric and oligomeric forms was automatically calculated using a routine developed in the commercial 3D image analysis software IMARIS-3D. The fraction of each state is expressed as a percentage. For example, if 30% of particles are monomers, it means that 30 out of 100 particles are in the monomeric form, while the remaining 70 particles are in aggregated states. Other image processing steps and RDF calculations were performed as described in detail in ref. 31. Examples of Cryo-ET tomograms and particle segmentation are presented in Fig. S14–S17.

### Small angle X-ray scattering (SAXS)

SAXS data shown in Fig. 4 and Fig. S10 were collected from synchrotron SAXS data at the beamline P12 operated by EMBL Hamburg at the PETRA III storage ring (DESY, Hamburg, Germany).^55^ We thank Dr Tobias Gräwert for assistance with beamline operation. The beam was operated at 10 keV, which corresponds to the wavelength of 0.124 nm. Sample-to-detector distance was set at 3 m. A Dectris photon counting Pilatus 6 M detector was used for the measurement. Approximately 25 μL of nanoparticle sample solutions were loaded into a quartz capillary (wall thickness: 50 μm; path length: 1.7 mm) using an automatic sample changer.^55^ The capillary was flushed sequentially with rapid-flow detergent, ethanol, and Milli-Q water prior to and between each sample measurement to prevent cross-contamination. Corresponding buffer solutions were measured before and after each sample to verify consistency and ensure no radiation damage occurred. Any spectra showing buffer intensity inconsistencies were excluded from further analysis.

SAXS data shown in Fig. S11 were measured from Xeuss 3.0 (Xenocs) at ETH with the help of Dr Thomas Weber, and those in Fig. S4 were measured using a Rigaku NanoMax-IQ setup at the Adolphe Merkle Institute (AMI) with the help of Dr Sandor Balog.

### Dielectric constant measurement

The dielectric constant was measured using a BI-870 Dielectric Constant Meter.

### AUC sedimentation velocity (SV) measurement

AUC-SV experiments were performed using a Beckman-Coulter XL-I analytical ultracentrifuge equipped with absorbance optics. Double-sector cells with path lengths of 12 mm or 3 mm were used, featuring sapphire windows and titanium center pieces. Nanoparticle dispersions (370 μL) with optical densities (OD) between 0.1 and 1 were loaded into the sample sector and water was injected in the reference sector. All measurements were conducted at 20 °C with rotor speeds set to acquire at least 40 data curves. Absorbance was monitored at 520 nm with a radial increment of 0.003 cm, and data acquisition was performed in continuous mode without delay. Sedimentation coefficient distributions, *C*(s), were analyzed using Sedfit v16.36 (https://www.analyticalultracentrifugation.com/).

## Author contributions

TM, QKO, and FS developed the ideas that are the basis for this paper. TM and PJS acquired all of the data. TM, JK, and KP were responsible for the treatment of all of the data. TM and FS wrote the manuscript; all authors contributed to its revisions.

## Conflicts of interest

All authors declare no conflicts of interest.

## Supplementary Material

NH-010-D5NH00260E-s001

## Data Availability

All data sets for this manuscript are available on Zenodo at https://doi.org/10.5281/zenodo.17038430. Supplementary information: Size distribution of AuNPs from TEM and SAXS. NMR results for evaluating purity and ligand composition of AuNPs. SAXS spectrum of AuNPs in water showing stability. NMR of 44OT AuNPs before and after annealing. AUC of 44OT AuNPs before and after annealing. Change of aggregation states due to proline and annealing. 2-Yukawa fitting of SAXS and comparison of Yukawa potential for allMUS AuNPs in H_2_O and in D_2_O. Cryo-ET tomographic reconstructions of nanoparticle samples. UV-Vis spectra of AuNPs. See DOI: https://doi.org/10.1039/d5nh00260e.
