# Supplementary material for: Proline stabilizes amphiphilic gold nanoparticles via hydrophobic interactions
Source: Nanoscale Horiz. 2025 Sep 16;10(11):3061–71. doi: 10.1039/d5nh00260e (PMC12439247; doi:10.1039/d5nh00260e)
Supplement: NH-010-D5NH00260E-s001 [file NH-010-D5NH00260E-s001.pdf]

Supplementary Information (SI) for Nanoscale Horizons.
This journal is © The Royal Society of Chemistry 2025

Supporting Information

Proline Stabilizes Amphiphilic Gold Nanoparticles via Hydrophobic Interactions

Ting Mao[1], Quy Khac Ong[1], Joachim Kohlbrecher[2], Ekaterina Poliukhina[1], Paulo Jacob Silva[1], Francesco Stellacci[1,3,4*]

1. Institute of Materials, Ecole Polytechnique Fédérale de Lausanne (EPFL), Lausanne, Switzerland

2. PSI Center for Neutron and Muon Sciences, Paul Scherrer Institute, Villigen PSI CH-5232, Switzerland

3. Bioengineering Institute, Ecole Polytechnique Fédérale de Lausanne (EPFL), Lausanne, Switzerland

4. Global Health Institute, Ecole Polytechnique Fédérale de Lausanne (EPFL), Lausanne, Switzerland

[*] Corresponding author: francesco.stellacci@epfl.ch

	Table of contents	Page NO.
Figure S1	Size distribution of AuNPs from TEM and SAXS	3, 4
Figure S2	NMR results of AuNPs purity	5, 6
Figure S3	NMR results of ligand composition of AuNPs	7
Figure S4	SAXS spectrum of AuNPs showing stability in water	8
Figure S5	NMR results of ligand stability of 44OT AuNPs before and after annealing	9
Figure S6	AUC results of stability of 44OT AuNPs before and after annealing	10
Figure S7	Proline effect in the aggregation states of the AuNPs shown in Figure 2(a-c)	11
Figure S8	Change in the aggregation states of 44OT AuNPs before and after annealing	12
Figure S9	Aggregation states comparison of allMUS, 21OT, 44OT AuNPs at similar number density	12
Figure S10	2-Yukawa fitting of SAXS measurement of allMUS AuNPs in D_2O	13
Figure S11	Comparison of attractive Yukawa potential of allMUS AuNPs in D_2O and H_2O	13
Figure S12	Comparison of repulsive Yukawa potential of allMUS AuNPs in D_2O and H_2O	14
Figure S13	Schematic of nanoparticles	14
Figure S14	Cryo-ET tomographic reconstructions of nanoparticle samples used to generate PMF curves in Fig. 2 (a-c)	15
Figure S15	Cryo-ET tomographic reconstructions of nanoparticle samples used to generate PMF curves in Fig. 2 (d-f)	16
Figure S16	Cryo-ET tomographic reconstructions of nanoparticle samples used to generate PMF curves in Fig. 3 (a-c)	17
Figure S17	Cryo-ET tomographic reconstructions of nanoparticle samples used to generate PMF curves in Fig. 3 (d-f)	18
Figure S18	UV-Vis spectra of gold nanoparticles	19

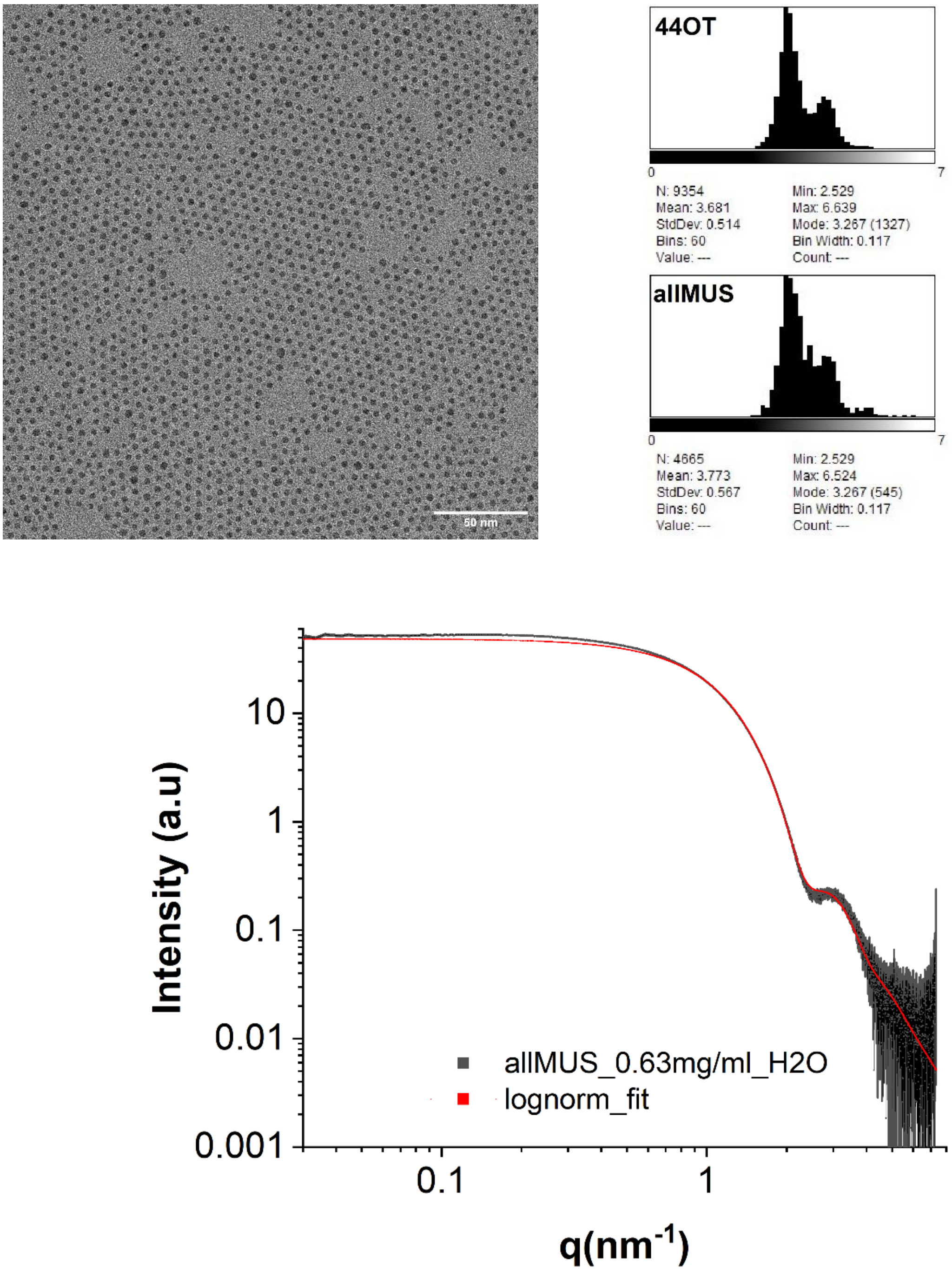

Figure S1. Size distribution characterization from TEM (top) and SAXS (bottom) of the allMUS AuNPs. On the top-left side is a TEM image of the as-synthesized oleylamine functionalized AuNPs before ligand exchange and graph on the top-right is a size (diameter) distribution histogram calculated for the allMUS (4665 counts) and 44OT(9354 counts) AuNPs. The bottom spectrum is SAXS spectrum of allMUS AuNPs in water (in black) at a very low concentration

(0.63mg/ml) showing no significant structure factor in the low q regime and its corresponding fitting (in red) from a log norm size distribution model of spherical particles.

Log-norm size distribution of the radius (X) of the particles were fitted from the lowest concentration of allMUS AuNPs in water. See equation below:

$$LogNorm\ (X, \mu, \sigma, p) = N \frac{1}{X^p} exp\left(-\frac{ln(^X/_\mu)^2}{2\sigma^2}\right)$$

Where N is the particle number density, σ is the width parameter, p is a shape parameter, μ is the location parameter. The fitted results are as follows

N	0.0555956
σ	0.154028
p	39.195
μ	4.41964

The mean of this log norm distribution is expressed as

$$X_{mean} = \mu e^{-\frac{1}{2}\sigma^2(2p-3)} = 1.807$$

Here we chose the mean as a fitted result of the representative size of the NPs, the fitted diameter should be d=2X~3.6 nm, which agrees with what is measured in TEM (~3.8/3.7 nm with a dispersity of ~0.5 nm).

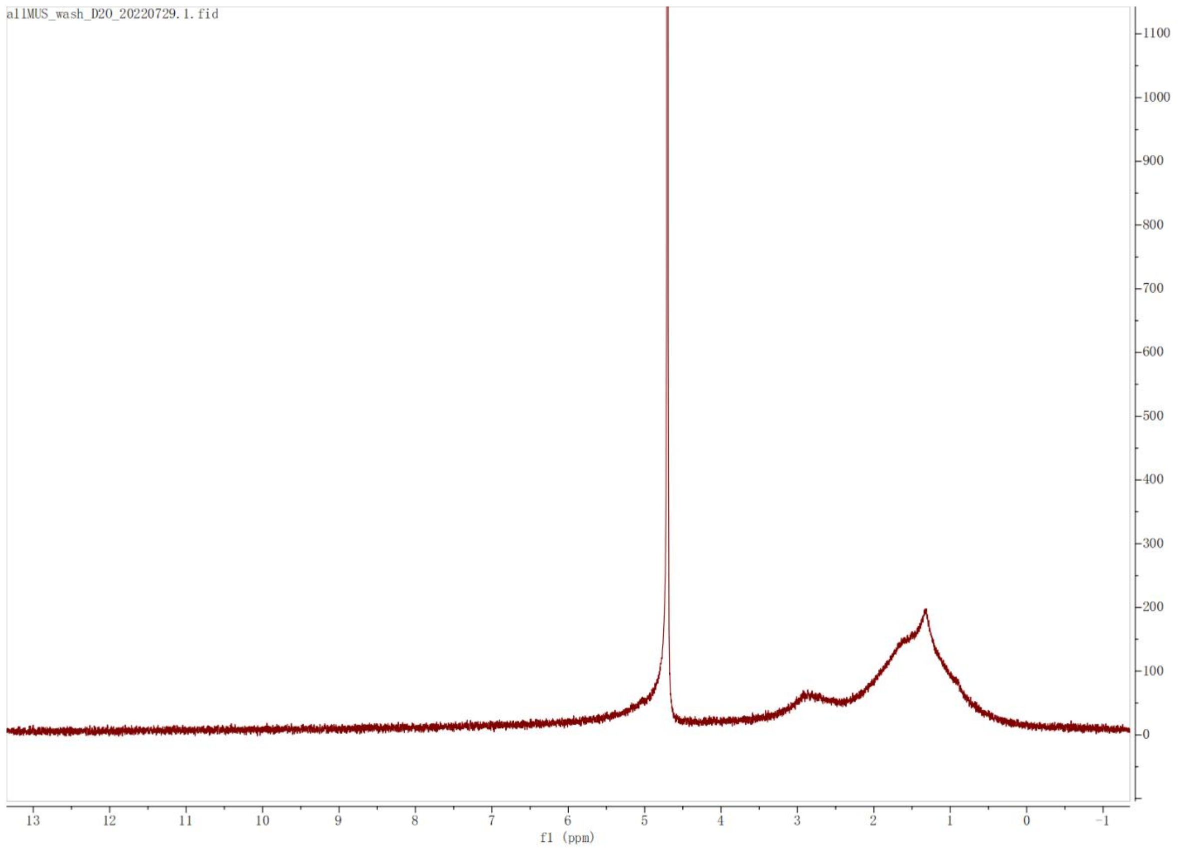
a11MUS_wash_D2O_20220729.1.fid
1100
1000
900
800
700
600
500
400
300
200
100
0
13
12
11
10
9
8
7
6
5
4
3
2
1
0
-1
f1 (ppm)

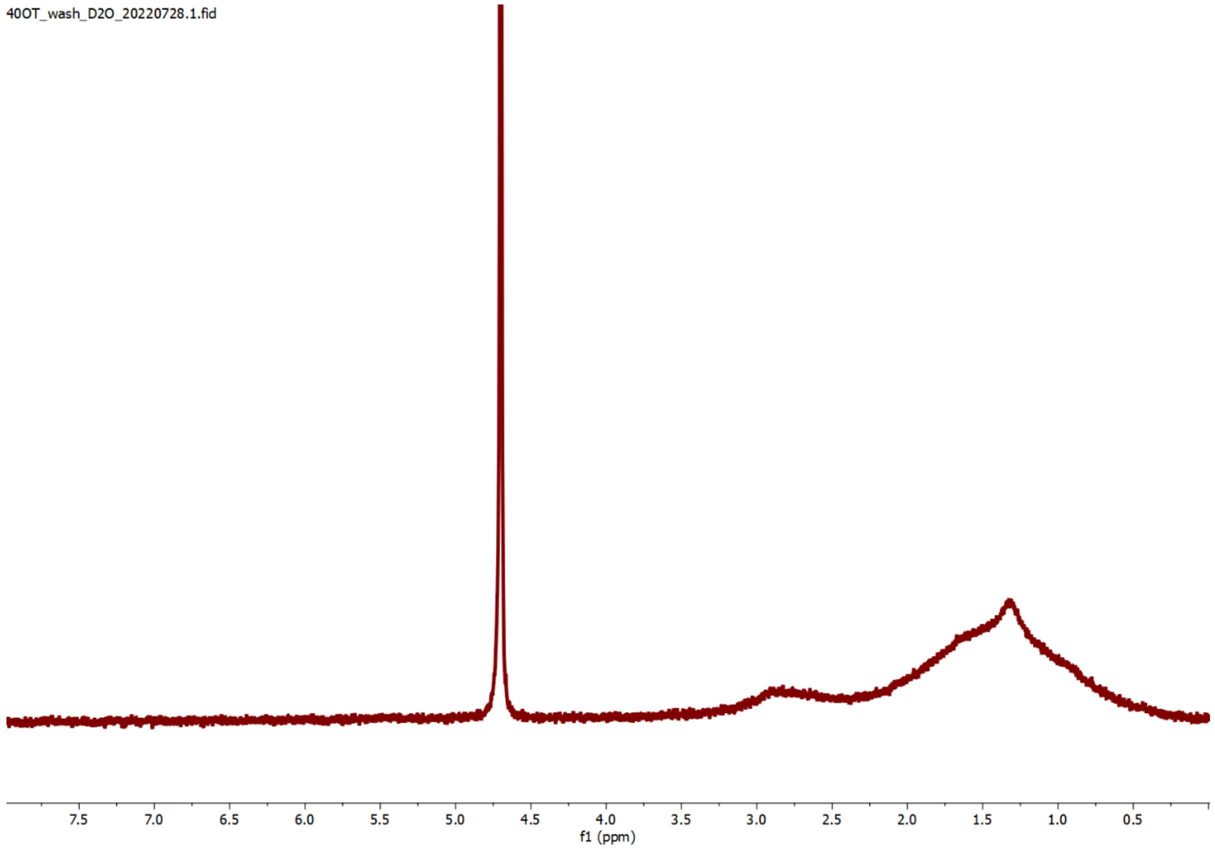
40OT_wash_D2O_20220728.1.fid
7.5
7.0
6.5
6.0
5.5
5.0
4.5
4.0
3.5
3.0
2.5
2.0
1.5
1.0
0.5
f1 (ppm)

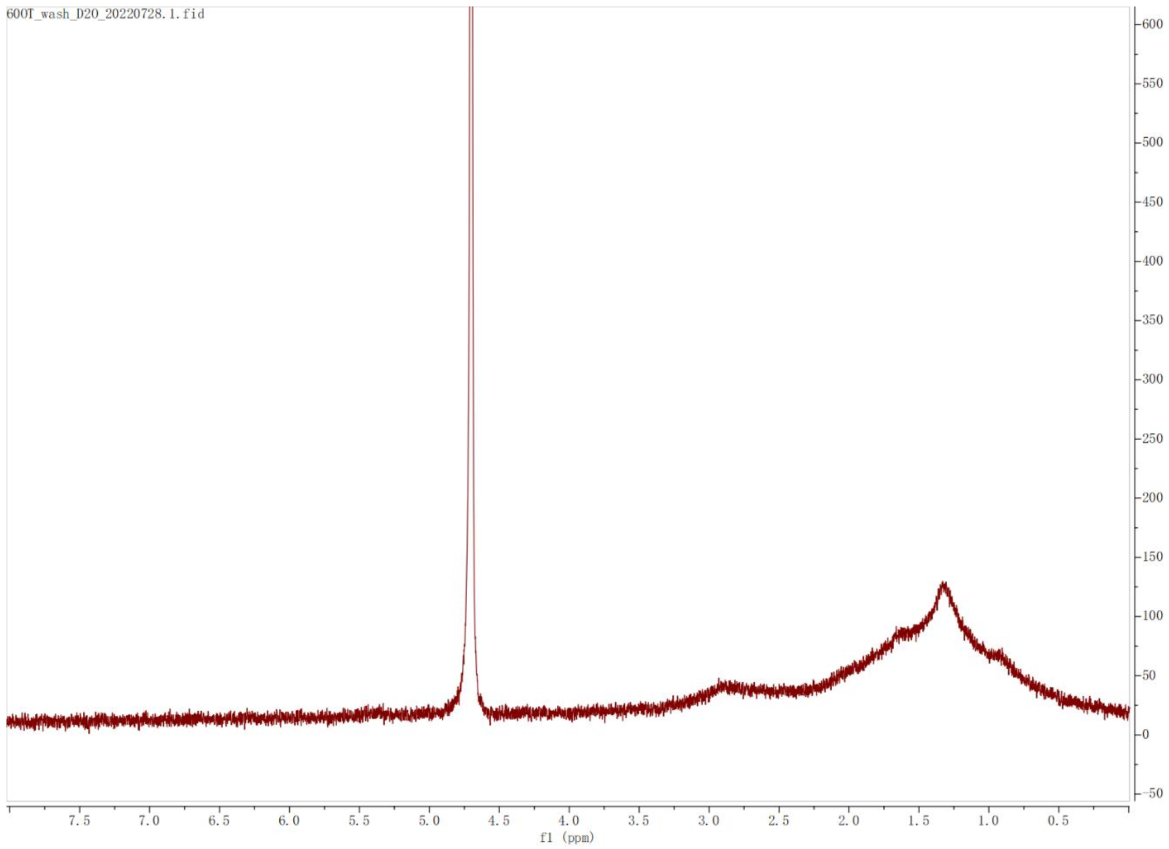

Figure S 2. In the order from top to bottom are the [1] H NMR of allMUS, 21OT, and 44OT AuNPs in D_2O, respectively. No sharp peak in the region of the free ligands (below 3.5 ppm of chemical shift) was observed.

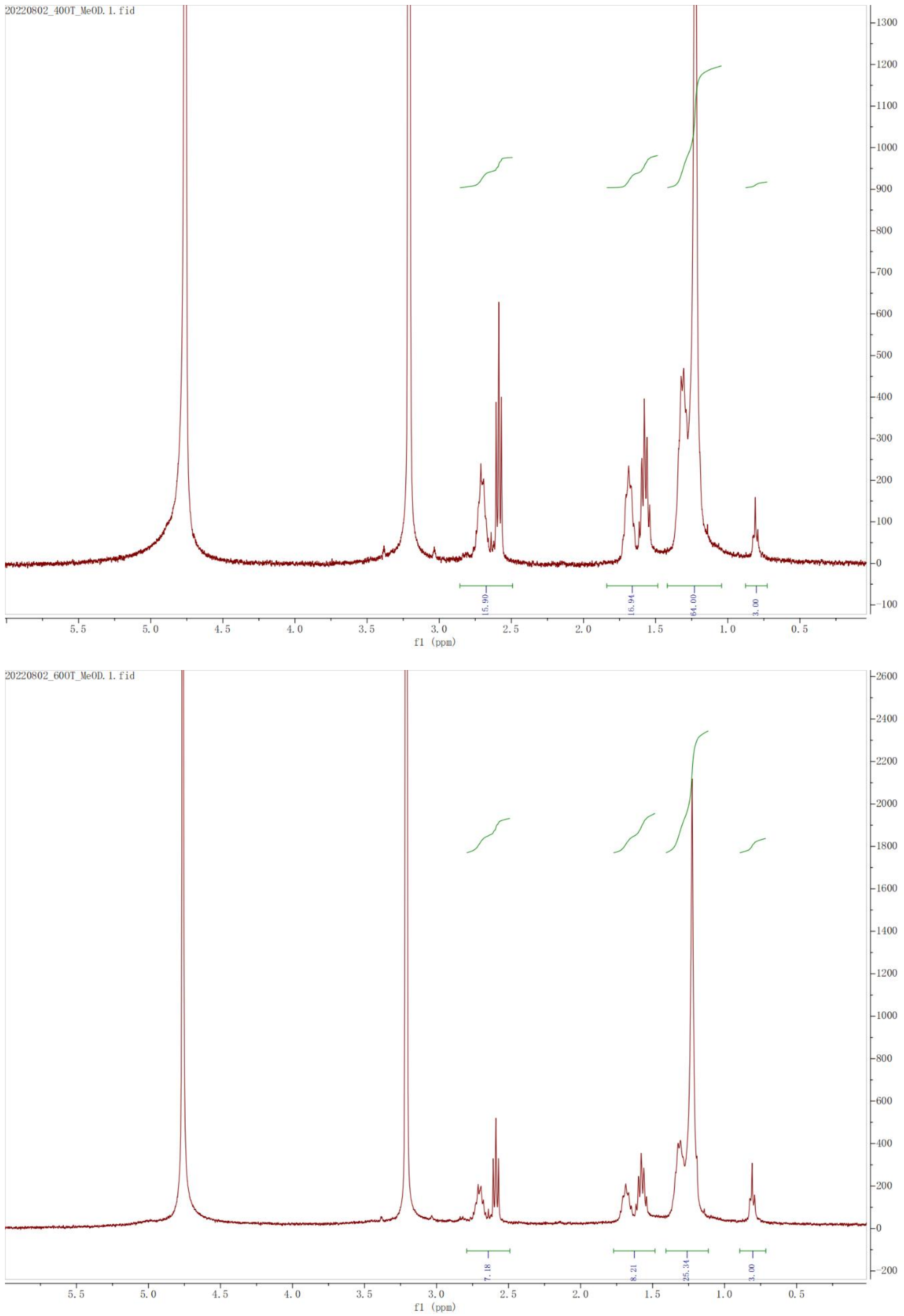

Figure S3. In the order from from top to bottom are respectively the ^{1}H NMR of the ligands of 21OT ((78.9±0.9)% of MUS and (21.1±0.9)% of OT) and 44OT ((56.5±4.3)% of MUS and

(43.5±4.3)% of OT) AuNPs in the mixture of iodine and MeOD. Integration values shown in the graphs were used for calculating composition of the mixed ligands.

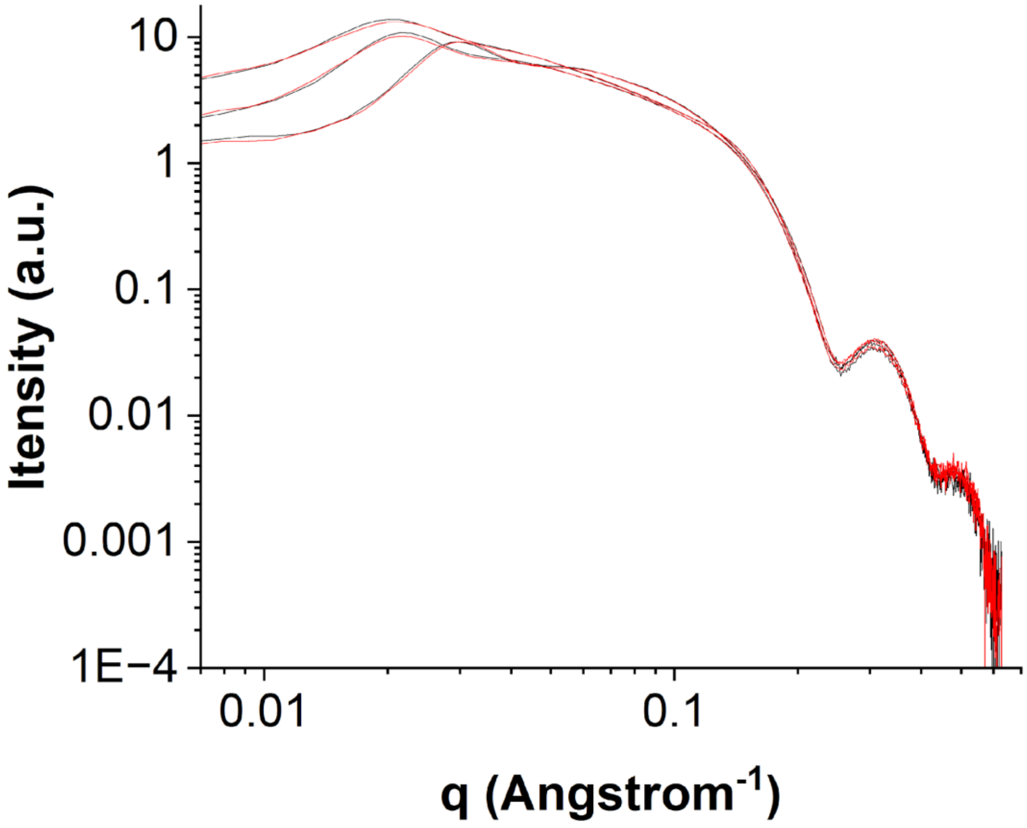

Figure S4. SAXS spectra of another batch of AuNPs with different relative composition (allMUS, 16OT and 30OT) of MUS and OT ligands in water, measured over a duration of 1 month. The red curves were obtained after 1 month from the reference time point (black curve curve).

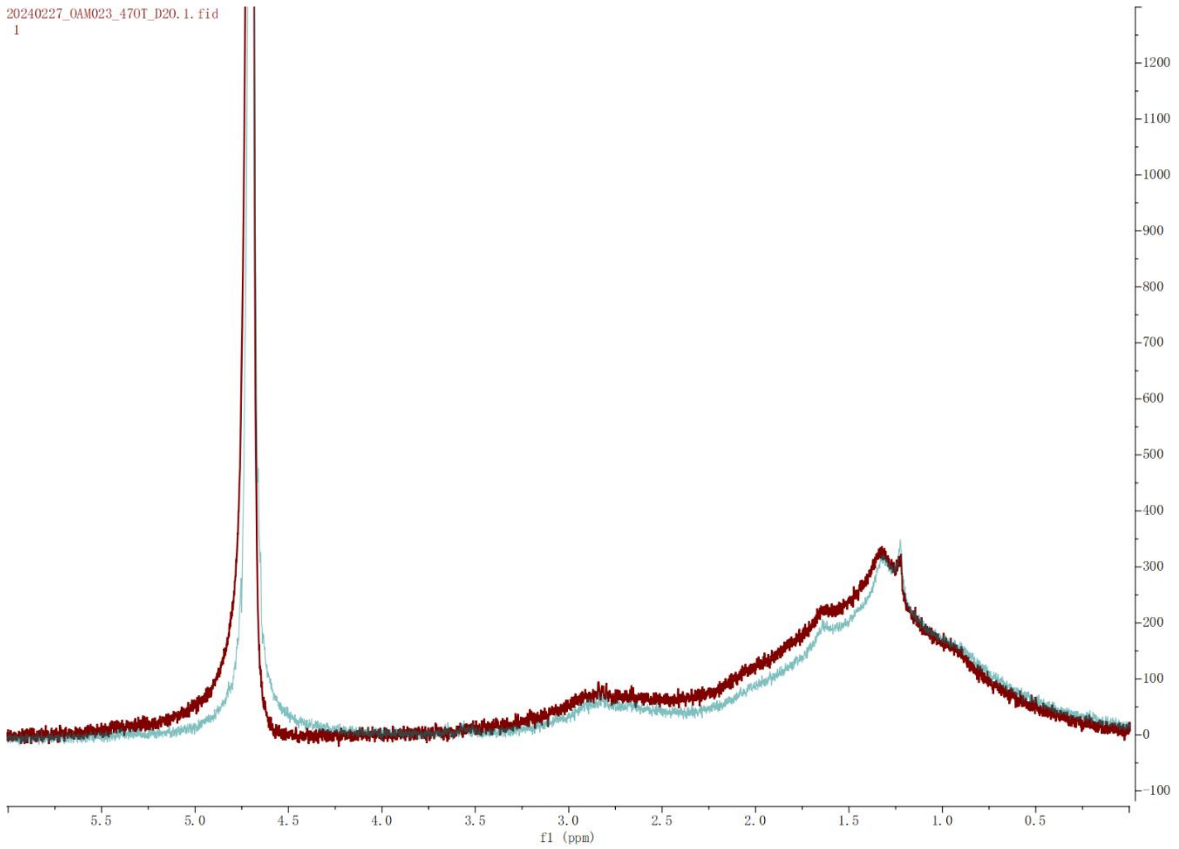

Figure S5. [1] H NMR comparison of the 44OT AuNPs before (dark red) and after annealing (blue) in D_2O.

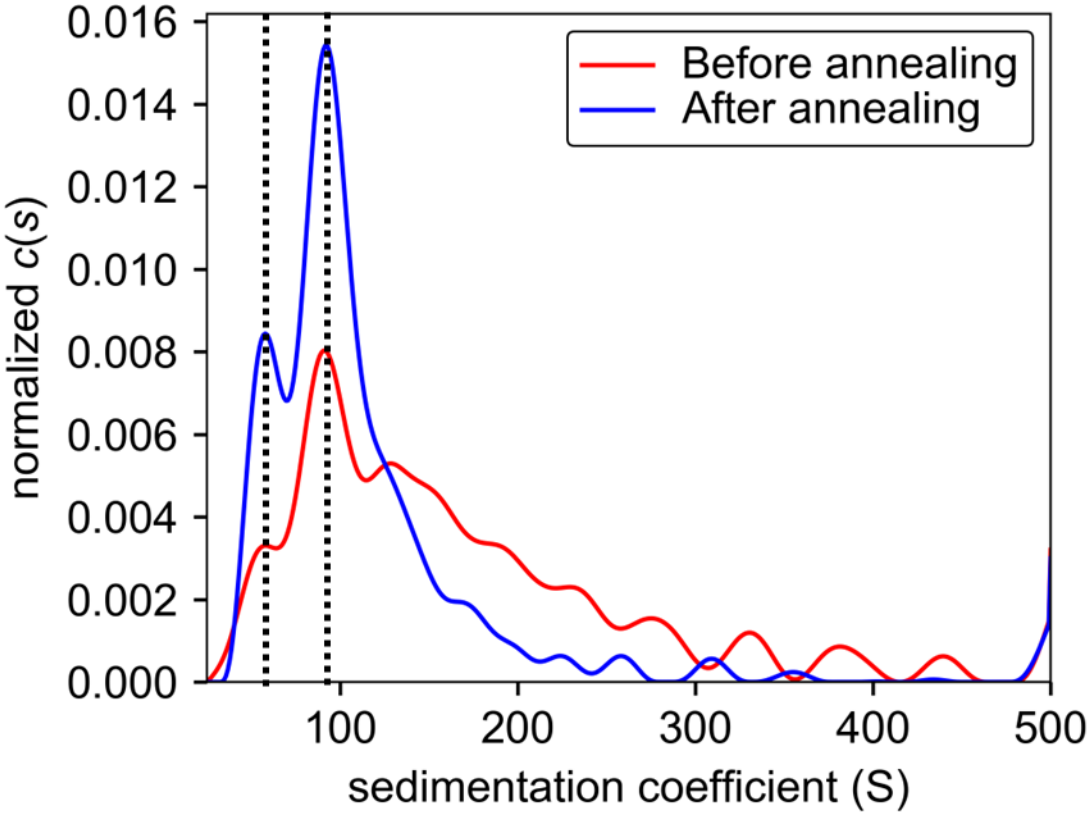

Figure S6. AUC-SV measurements of the 44OT AuNPs before (red) and after (blue) annealing, showing no shift in the sedimentation coefficient of the monomer populations.

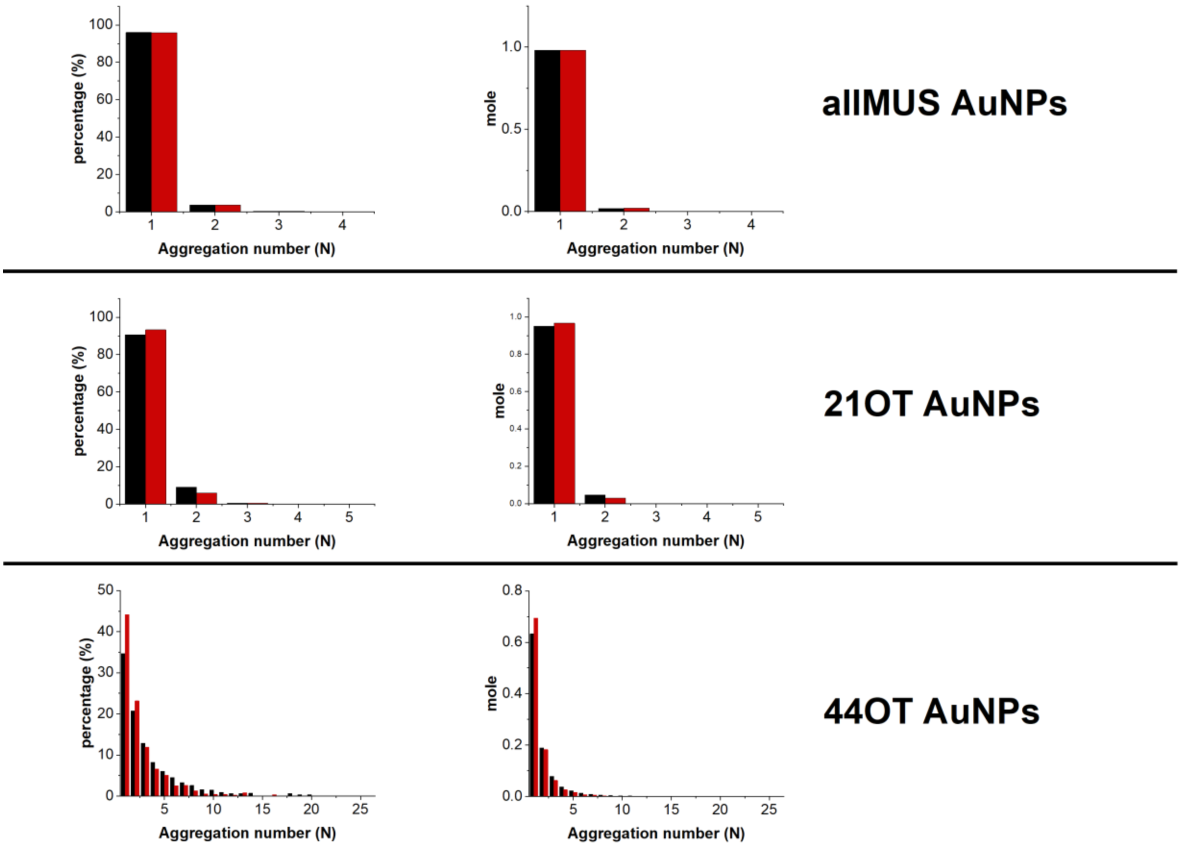

Figure S7. Aggregation distribution of the same AuNPs as in Figure 2(a-c), with different hydrophobicity and their small molecule effect (addition of 2M proline). Red indicates with proline and black indicates without. Concentration conditions presented are the same in terms of number density as described in Figure 2(a-c).

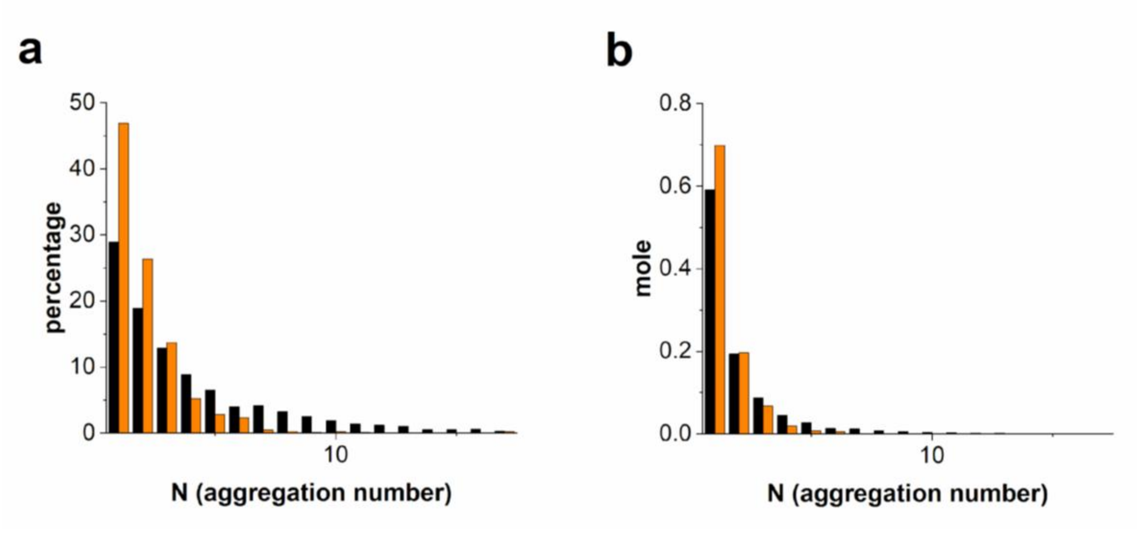

Figure S8. Aggregation distribution of 44OT AuNPs before and after annealing, at relatively the same number density: aggregation 44OT in water (11.35×10^{22} (black)), annealed 44OT for 24 hrs (11.6×10^{22} (orange)).

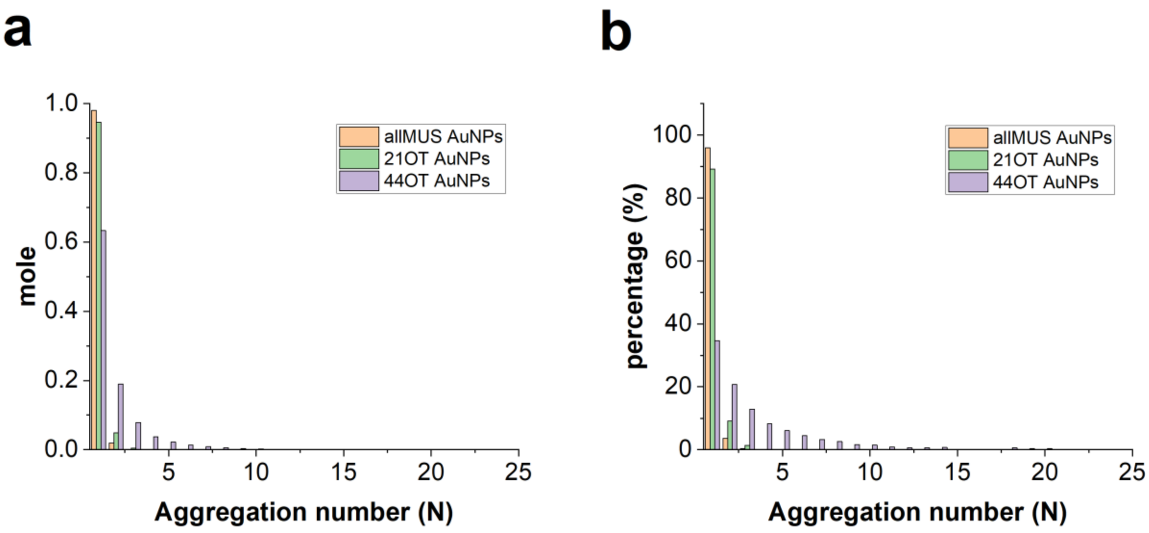

Figure S9. Aggregation distribution (mole(a) and percentage(b) distribution) of AuNPs in different hydrophobicity: (in units of $particles/m^3$):allMUS 5.28×10^{22} (sandy brown), 21OT of 5.49×10^{22} (green), 44OT 5.14×10^{22} (purple)

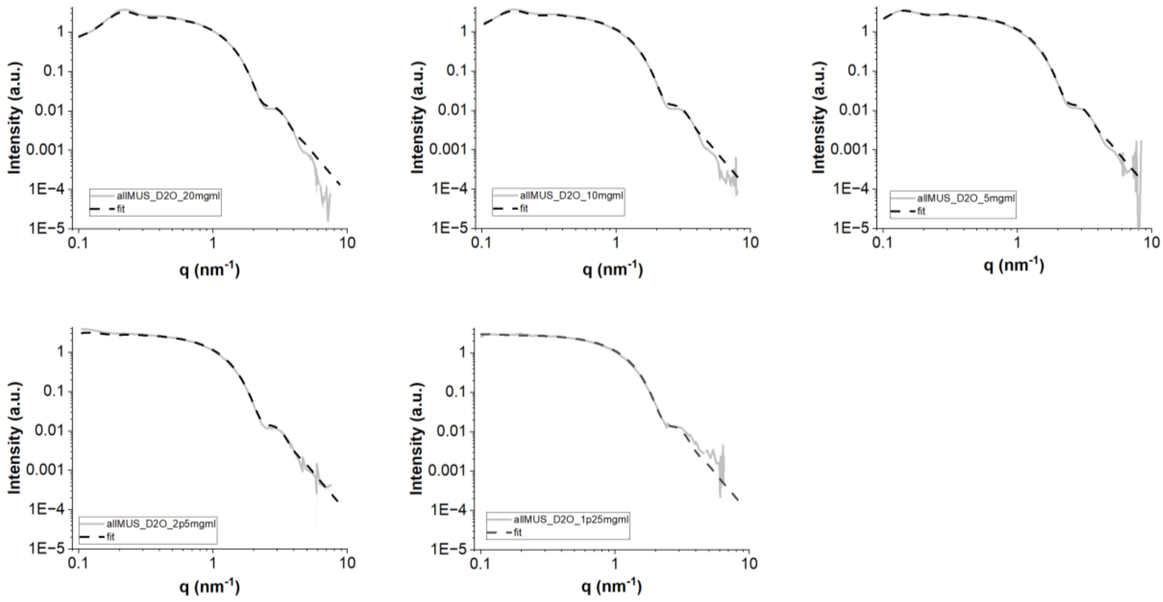

Figure S10. SAXS spectra of allMUS in heavy water and their fit with a 2-Yukawa potential. From left to right, top to bottom is correspondingly the measured SAXS spectrum (in solid grey) of allMUS AuNPs in heavy water in different concentrations (20mg/ml, 10mg/ml, 5mg/ml, 2.5mg/ml and 1.25mg/ml relatively) and their fit with the 2-Yukawa model (in dashed black).

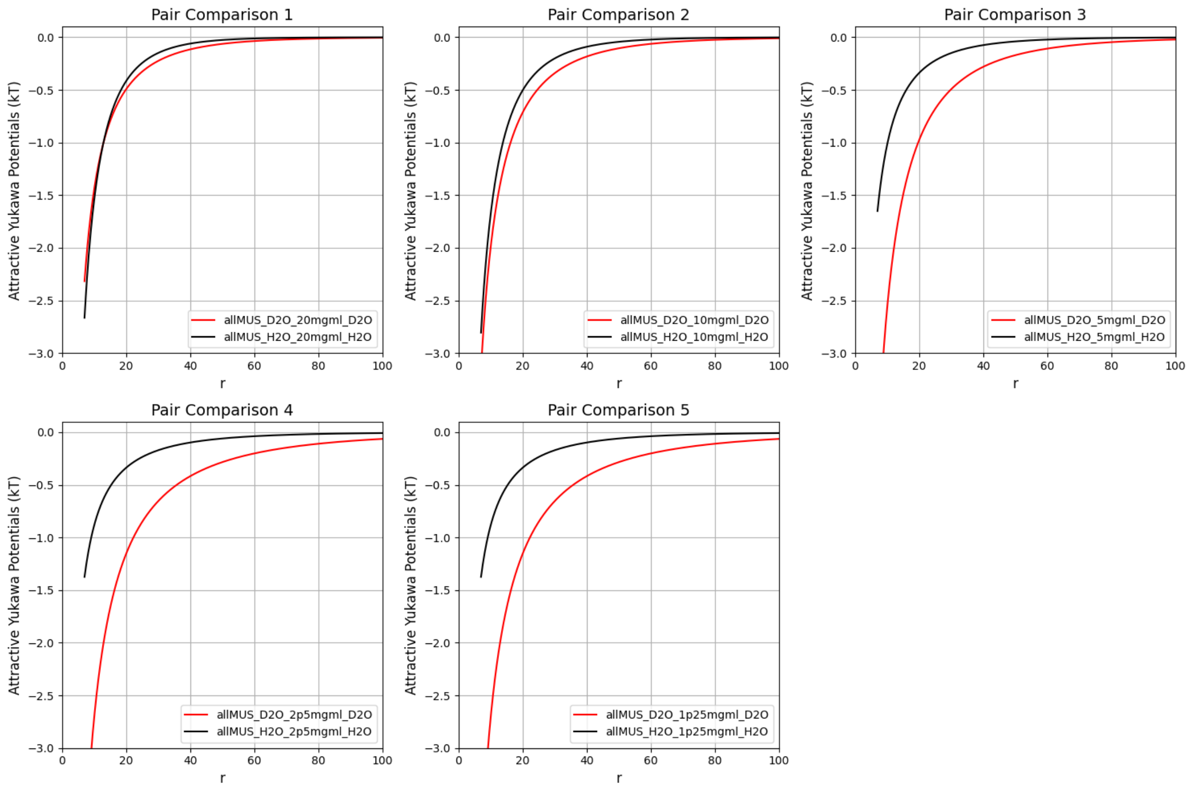

Figure S11. Comparison of attractive Yukawa potential curves of allMUS AuNPs in water (black) and heavy water (red), from 2-Yukawa fit. From left to right, top to bottom is correspondingly the concentration series of 20mg/ml, 10mg/ml, 5mg/ml, 2.5mg/ml and 1.25mg/ml.

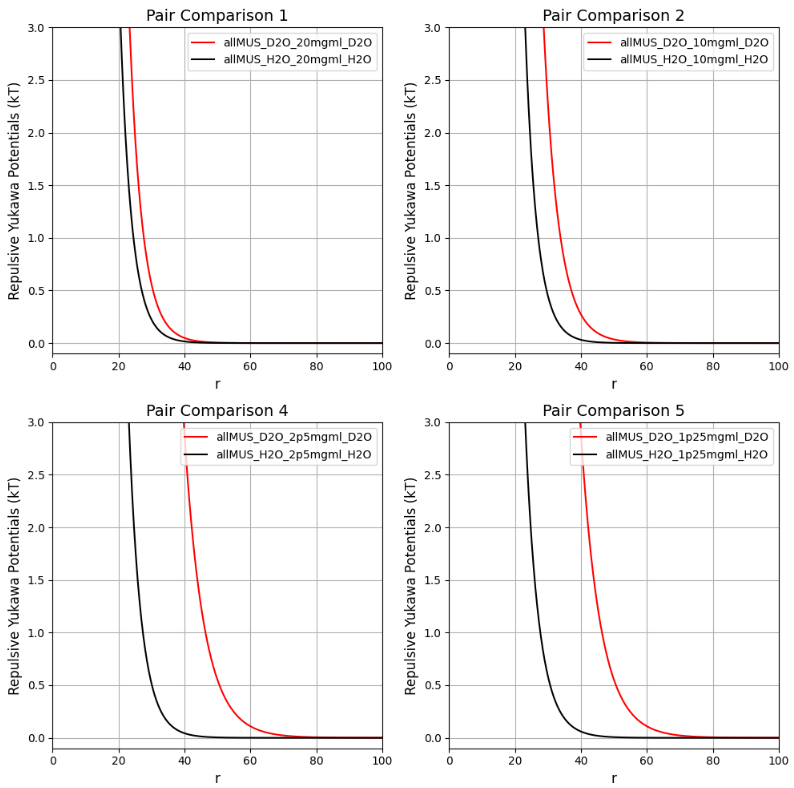

Figure S12. Comparison of repulsive Yukawa potential curves of allMUS AuNPs in water (black) and heavy water (red), from 2-Yukawa fit. From left to right, top to bottom is correspondingly the concentration series of 20mg/ml, 10mg/ml, 5mg/ml, 2.5mg/ml and 1.25mg/ml.

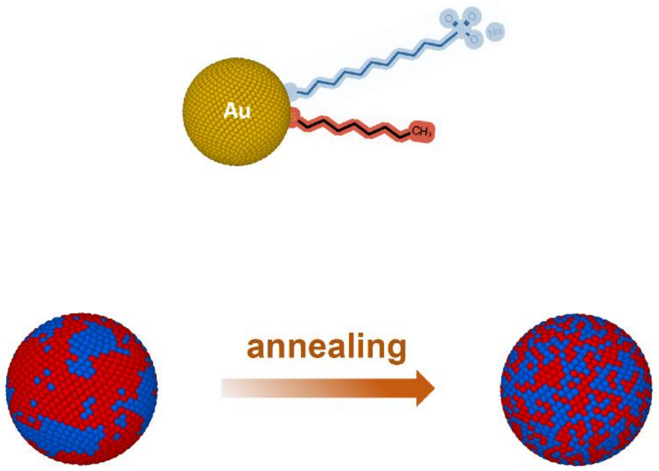

Figure S13. Schematic representation of amphiphilic gold nanoparticles and ligand reorganization upon thermal annealing. (Top) Gold nanoparticle (Au) functionalized with a mixed monolayer of hydrophilic 11-mercaptoundecane sulfonate (MUS, blue) and hydrophobic 1-octanethiol (OT, red) ligands. (Bottom) Effect of thermal annealing on ligand shell morphology showing the transformation from phase-separated domains (left) to randomly mixed ligands (right), resulting in smaller hydrophobic patches and enhanced hydrophobic character of the nanoparticle surface.

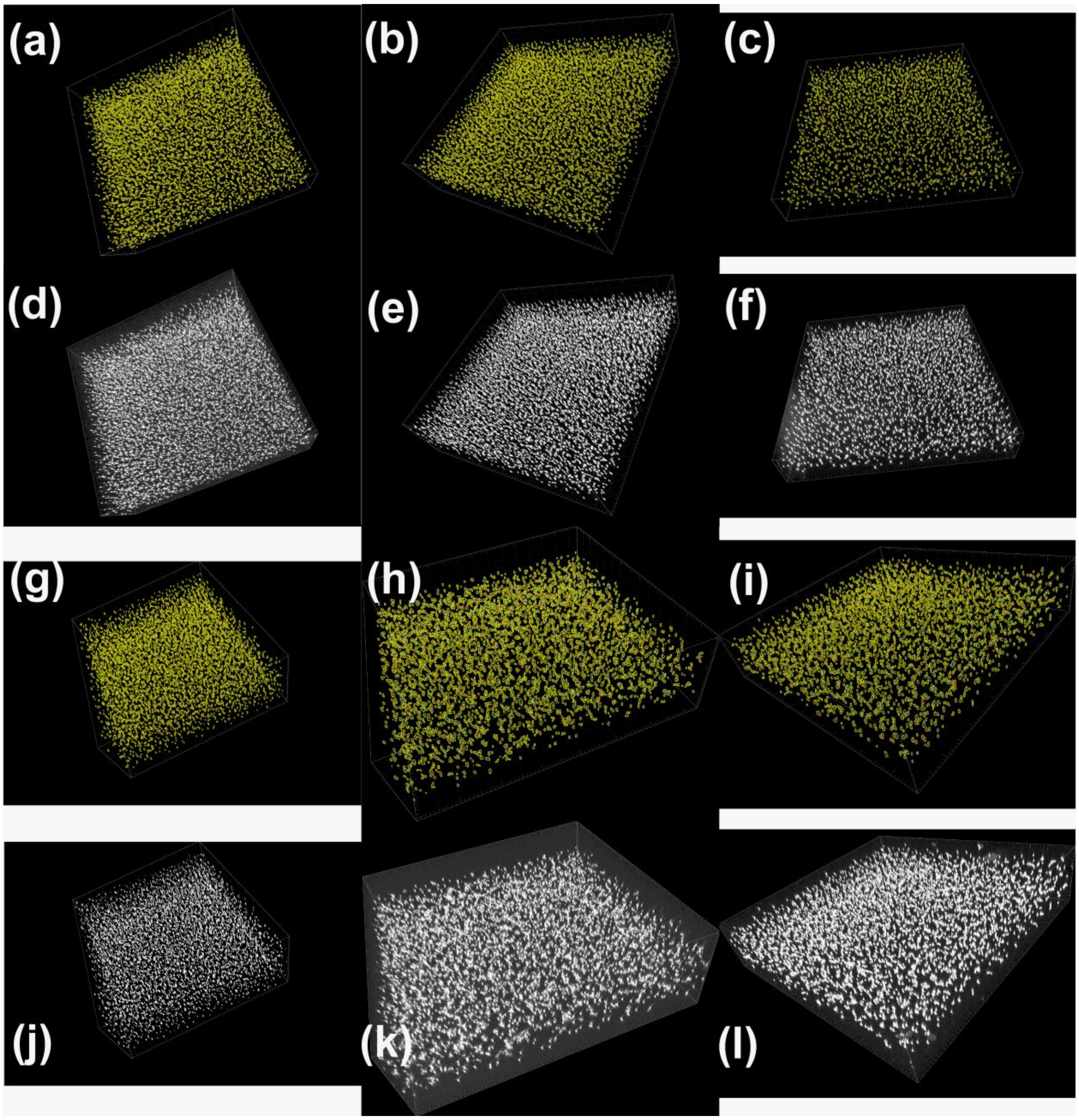

Figure S14 Cryo-ET tomographic reconstructions of nanoparticle samples used to generate PMF curves in Fig. 2 (a-c). Top panels (a-c, g-i): 3D surface segmentations of detected nanoparticles (yellow). Bottom panels (d-f, j-l): Corresponding raw tomographic data (grayscale). (a,d) allMUS AuNPs in H_2O without proline, (b,e) allMUS AuNPs in H_2O with 2M proline, (c,f) 21%OT AuNPs in H_2O without proline, (g,j) 21%OT AuNPs in H_2O with 2M proline, (i,l) 44%OT AuNPs in H_2O without proline, (k,l) 44%OT AuNPs in H_2O with 2M proline.

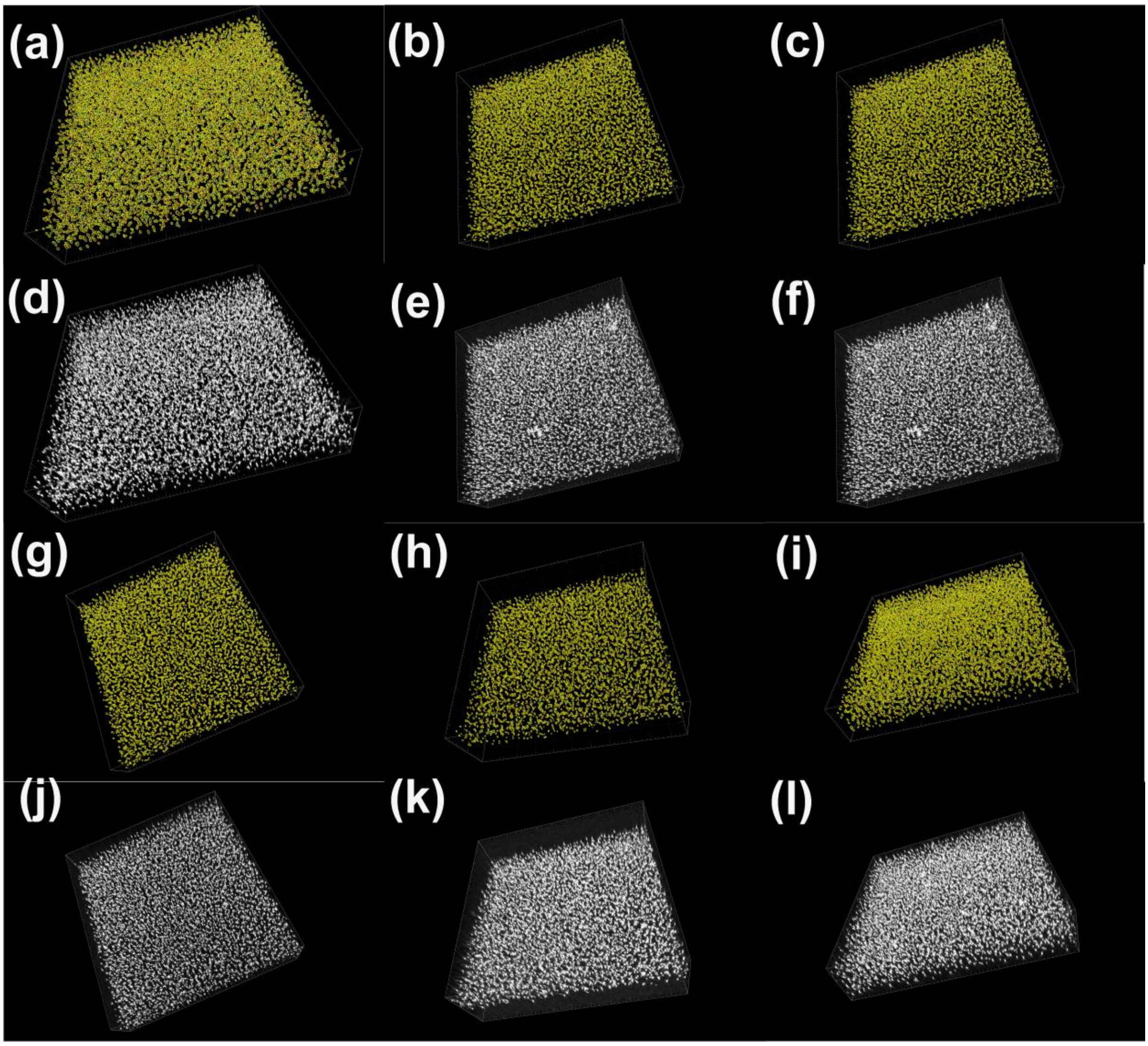

Figure S15 Cryo-ET tomographic reconstructions of nanoparticle samples used to generate PMF curves in Fig. 2 (d-f). Top panels (a-c, g-i): 3D surface segmentations of detected nanoparticles (yellow). Bottom panels (d-f, j-l): Corresponding raw tomographic data (grayscale). (a,d) 44%OT AuNPs in H_2O without annealing, (b,e) 44%OT AuNPs in H_2O after 24 hrs annealing, (c,f) the same annealed 44%OT AuNPs in H_2O, (g,j) annealed 44%OT AuNPs in D_2O, (h,k) annealed 44%OT AuNPs in H_2O, (i,l) annealed 44%OT AuNPs in H_2O with 2M proline.

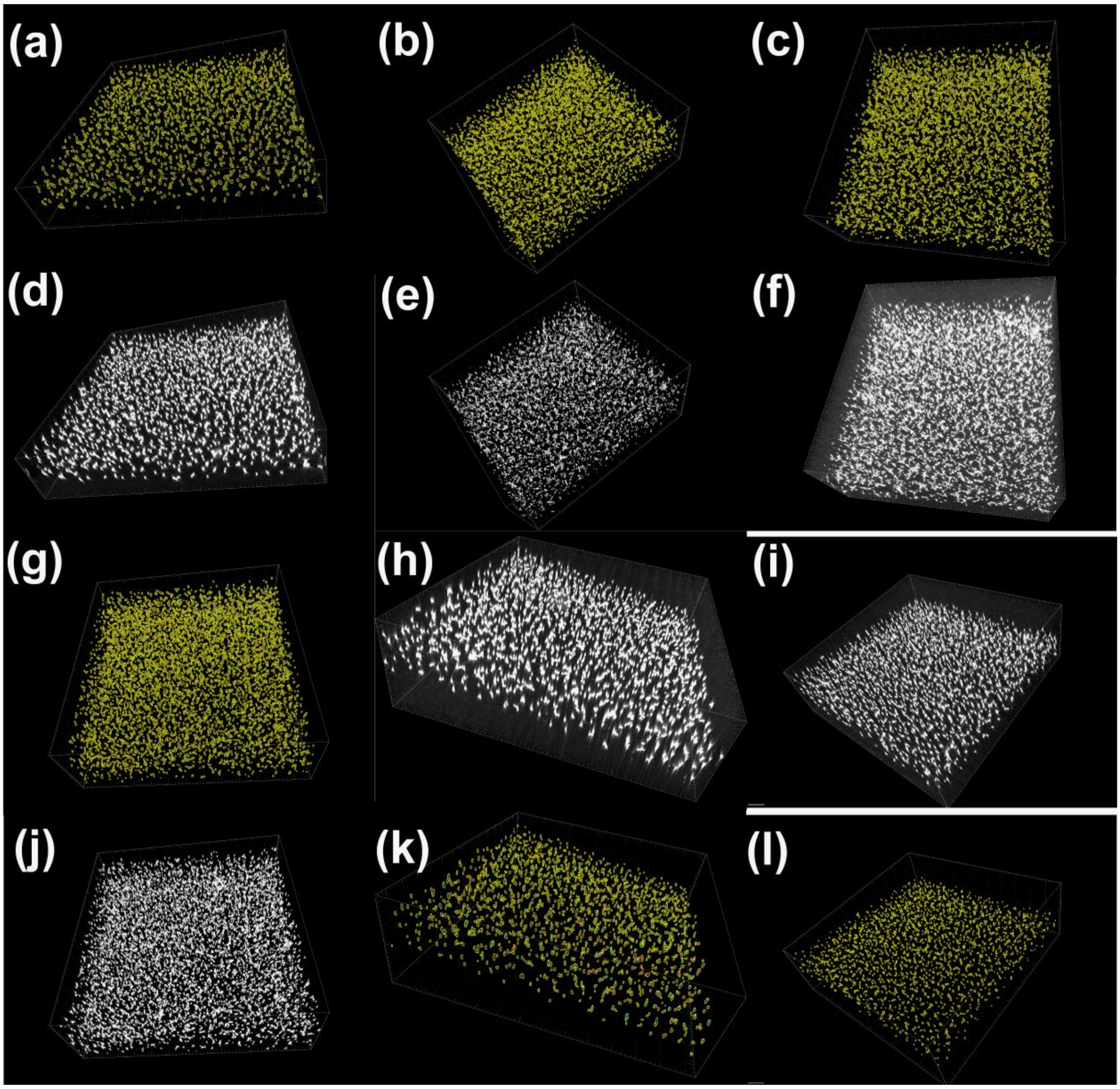

Figure S16 Cryo-ET tomographic reconstructions of nanoparticle samples used to generate PMF curves in Fig. 3 (a-c). Top panels (a-c, g-i): 3D surface segmentations of detected nanoparticles (yellow). Bottom panels (d-f, j-l): Corresponding raw tomographic data (grayscale): (a,d) 44%OT AuNPs in H_2O, (b,e) 44%OT AuNPs in D_2O, (c,f) 44%OT AuNPs in D_2O, (g,j) 44%OT AuNPs in D_2O with 2M proline, (h,k) 44%OT AuNPs in H_2O, (i,l) 44%OT AuNPs in H_2O with 2M proline.

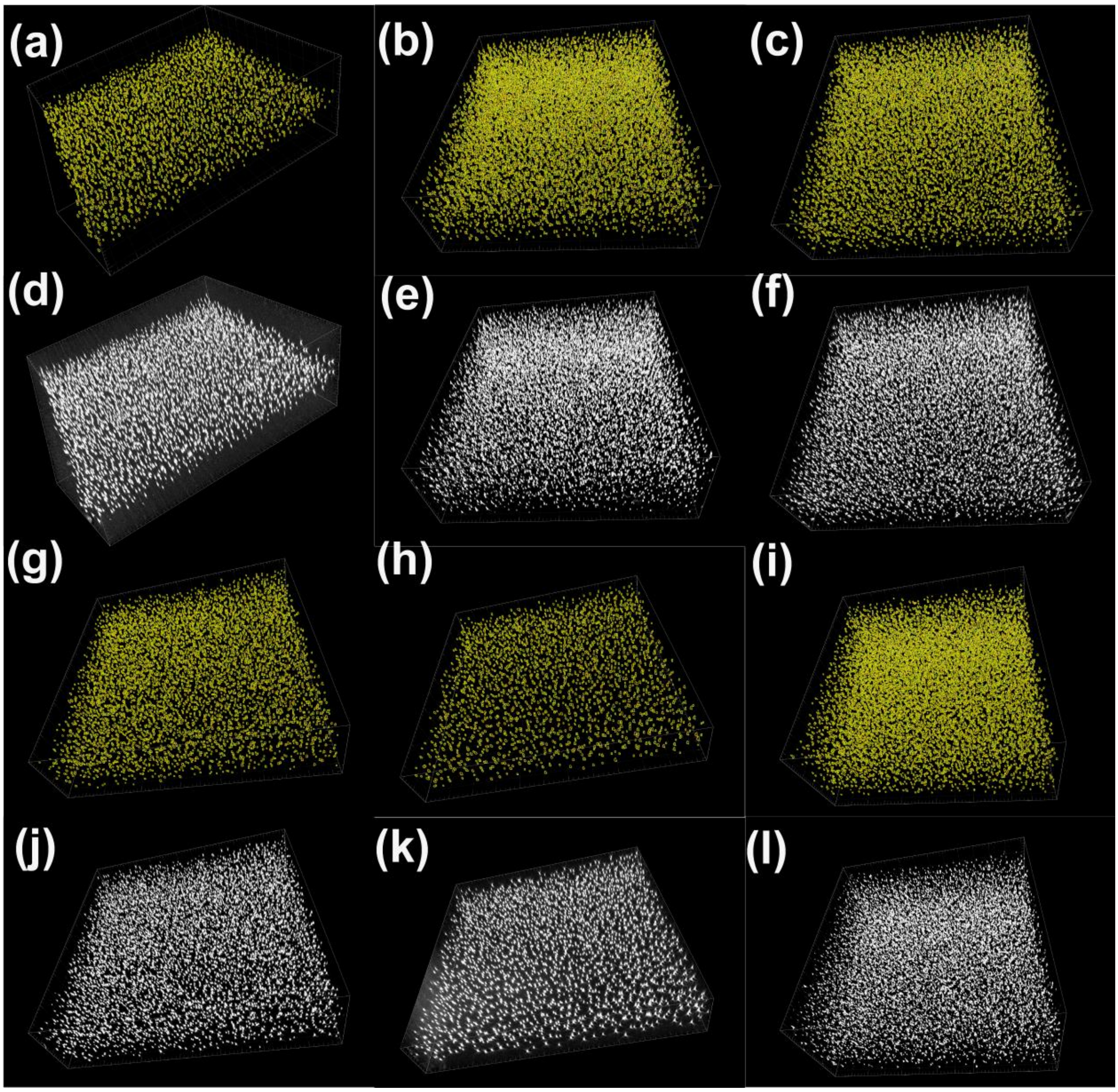

Figure S17 Cryo-ET tomographic reconstructions of nanoparticle samples used to generate PMF curves in Fig. 3 (d-f). Top panels (a-c, g-i): 3D surface segmentations of detected nanoparticles (yellow). Bottom panels (d-f, j-l): Corresponding raw tomographic data (grayscale). (a,d) 21%OT AuNPs in H_2O, (b,e) 21%OT AuNPs in D_2O, (c,f) 21%OT AuNPs in D_2O, (g,j) 21%OT AuNPs in D_2O with 2M proline, (h,k) 21%OT AuNPs in H_2O, (i,l) 21%OT AuNPs in H_2O with 2M proline.

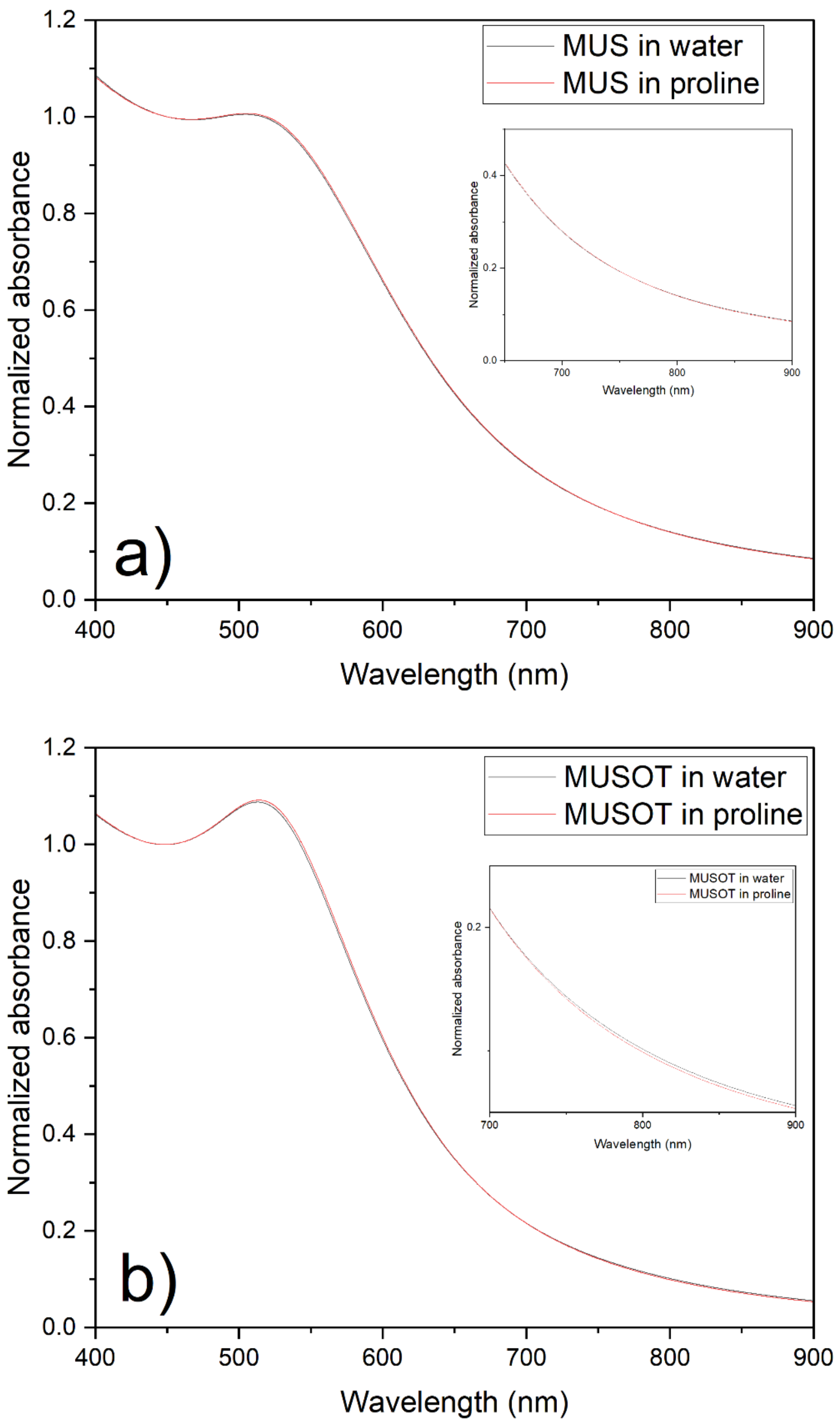

Figure S18. UV-Vis spectra of gold nanoparticles in water and in water containing proline. (A) allMUS gold nanoparticles; (B) MUSOT gold nanoparticles. For both types of nanoparticles, spectra were measured using a stock solution that was diluted 1:1 either with water or with water containing 3 M proline. This resulted in final dispersions of gold nanoparticles in pure water or in 1.5 M proline. All spectra were normalized to their absorbance at 450 nm. Insets contain a zoomed-in view of the spectra at high wavelengths.
